# The importance of finite temperature and vibrational sampling in the absorption spectrum of a nitro-functionalized Ru(ii) water oxidation catalyst†

**DOI:** 10.1039/d1cp02748d

**Published:** 2021-08-04

**Authors:** Anna M. Wernbacher, Leticia González

**Affiliations:** Institute of Theoretical Chemistry, Faculty of Chemistry, University of Vienna 1090 Vienna Währinger Straße 17 Austria anna.wernbacher@univie.ac.at leticia.gonzalez@univie.ac.at

## Abstract

Consideration of finite temperature and vibrational motion can be an essential component for accurate simulations of absorption spectra. Here we use finite-temperature Wigner phase-space sampling to investigate the intense absorption of the water oxidation catalyst Ru(dppip-NO_2_) in the visible (vis) region. The influence of vibrational and torsional motions as well as temperature effects are addressed for the different protonation forms of the pH-sensitive dppip-NO_2_ ligand of the catalyst. Excitations to the nitrophenyl group and π-system of dppip-NO_2_, which characterize the absorption band in the equilibrium spectra, experience energy shifts and a significant decrease in oscillator strength when nuclear motion is considered. The importance of excitations to the nitrophenyl group for the vis band is reduced in the spectra computed from the 300 K ensembles, which feature broad distributions of the corresponding dihedral angles. The effects of vibrational sampling on the absorption spectra may be attributed to nitrophenyl and, in particular, to NO_2_ torsional motions. We expect finite temperature and vibrational sampling to be important for simulating the absorption spectra of other transition metal complexes with flexible ligands or nitro-aromatic motifs.

## Abstract 2

The influence of vibrational effects on the absorption spectra of a Ru water oxidation catalyst are shown: comparison of a nuclear ensemble approach (red, Wigner sampling) with spectrum based on a single equilibrium geometry (blue).

## Graphical abstract

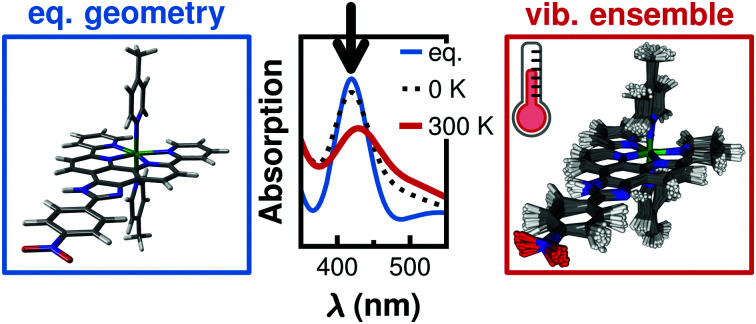

## 1 Introduction

**Fig. 1:**
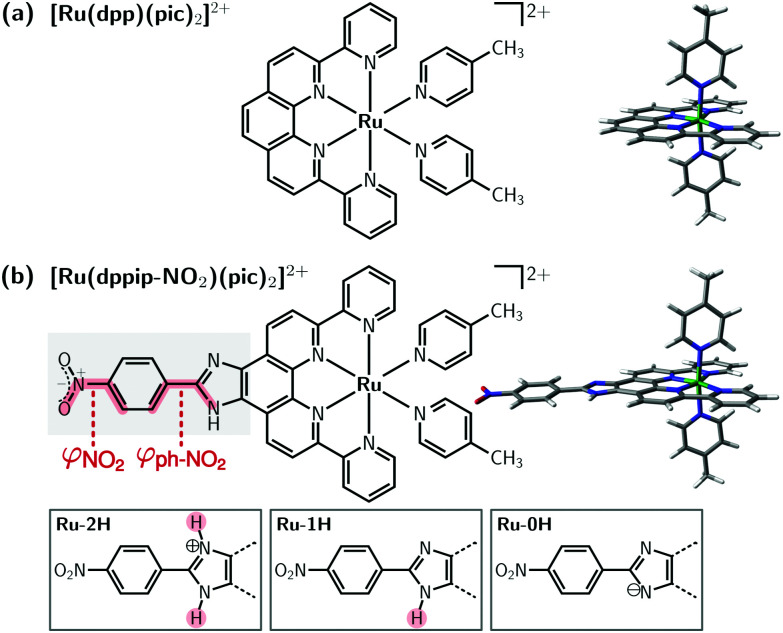
Structure of (a) [Ru(dpp)(pic)_2_]^2+^ “Ru(dpp)” and (b) [Ru(dppip-NO_2_)(pic)_2_]^2+^ “Ru(dppip-NO_2_)”. The NO_2_ dihedral angle (*φ*_NO_2__) and nitrophenyl dihedral angle with respect to the dppi ring system (*φ*_ph–NO_2__) are highlighted in red; the imidazole group of the dppip-NO_2_ ligand with two (2H), one (1H), and zero protons (0H) is indicated at the bottom.

The photophysical and redox properties of ruthenium complexes render them interesting to use in dye-sensitized solar cells,^1^ photocatalytic water splitting,^2,3^ or in photoredox catalysis,^3–5^ to name a few applications. For example, mononuclear complexes such as [Ru(dpp)(pic)_2_]^2+^ (“Ru(dpp)”, dpp = di-pyridyl-phenanthroline, pic = 4-picoline; see Fig. 1a),^6,7^ are appealing catalysts for the water oxidation reaction and exhibit a reduced complexity compared to polynuclear ruthenium compounds.

Recently, we presented^8^ a modified water oxidation catalyst based on Ru(dpp) decorated with an electron-withdrawing nitrophenyl group (ph-NO_2_) and a pH-sensitive imidazole (im) motif on the equatorial ligand dppip-NO_2_, [Ru(dppip-NO_2_)(pic)_2_]^2+^ (“Ru(dppip-NO_2_)”, dppip-NO_2_ = 2-(4-nitrophenyl)-6,9-di(pyridin-2-yl)-1*H*-imidazo[4,5-*f*]1,10-phenanthroline; see Fig. 1b). This functionalized ligand may serve as starting point for further chemical modifications such as introduction of an azide or amide. Interestingly, this modification significantly affects the absorption spectrum of the Ru-complex compared to the parent compound Ru(dpp). While the photophysical properties of Ru(dpp) could previously be tuned by modifying the axial pyridine-type ligands (pic),^6^ modifications of the dpp-based ligand did not significantly change the absorption bands.^7^ The functionalized dppip-NO_2_ ligand, however, gives rise to a strong absorption in the visible (vis) region, which can furthermore be tuned by (de)protonation of the imidazole moiety, as shown by the pronounced pH-sensitivity of the experimental UV-vis spectra.^8^ In contrast, the parent complex Ru(dpp) absorbs in the UV region except for weak metal-to-ligand charge transfer bands in the vis energy range.

Spectroscopic or spectro-electrochemical investigations are often performed routinely, ideally as *in situ* or *operando* experiments, in order to characterize the complexes and to gain insight into the working mode of the catalysts. The combination of computational studies with UV-vis absorption spectroscopic experiments can further provide information about the relevant electronically excited states as well as help determine the spectroscopic signatures of the catalytic system. While the calculation of electronic absorption spectra is also frequently done for medium-sized organic chromophores,^9–11^ an accurate description in the case of transition metal complexes is not without challenges. These are mainly related to the high density of states and the often complex electronic structure, among other factors.^12,13^ One particular aspect that we would like to focus on here is the additional difficulty that flexible functional groups pose – as in the modified ligand in Ru(dppip-NO_2_).

When flexible groups are present, it has been realized that a static description of electronic absorption spectra relying only on computing vertical excitation energies at the ground-state equilibrium (minimum-energy) geometry, may not satisfactorily describe the spectra compared to experiments.^14^ Consideration of the vibrational motion of the nuclei leads to a better description of the spectral line shapes and the position of the absorption maxima.^14–17^ Nuclear vibrational effects can even change the character of the absorption bands, as it has been shown for some nitro-aromatic compounds.^18–20^ Despite essential, only a few studies^21–24^ take into account the influence of vibrational sampling on the simulation of absorption spectra of systems in general and of transition metal complexes in particular.

In this work, we investigate the absorption spectra of Ru(dppip-NO_2_) addressing the influence of the chemical modification of the ligand and of ligand (de)protonation, paying particular attention to vibrational sampling. In particular, we would like to find out how vibrational sampling influences the absorption spectra of the different protonated forms of Ru(dppip-NO_2_) and whether the previously reported quenching of the charge transfer character by vibrational effects in organic nitro-aromatic compounds,^18–20^ here translated into the torsional motion within the functionalized ligand, affects the absorption.

## 2 Computational methods

As Ru(dppip-NO_2_) has a pH-sensitive imidazole moiety on the equatorial dppip-NO_2_ ligand, we investigated structures with two protons (2H-Ru(dppip-NO_2_)), one proton (1H-Ru(dppip-NO_2_)), and without protons (0H-Ru(dppip-NO_2_)) on the imidazole group using density functional theory (DFT). When referring to the different protonation forms of the Ru(dppip-NO_2_) complex, we will use the abbreviations Ru-0H (zero protons at im), Ru-1H (one proton at im, neutral form of the dppip-NO_2_ ligand), and Ru-2H (two protons at im) as indicated in Fig. 1b. The geometries of the different protonated species of the Ru(dppip-NO_2_) complexes, as well as Ru(dpp) for comparison, were optimized with the B3LYP^25–27^ density functional and the D3BJ^28,29^ dispersion correction in combination with the def2-TZVP^30^ basis set and the corresponding effective core potential (def2-ECP)^31^ on Ru. Further, the calculations used the RIJCOSX^32–34^ approximation for computational efficiency^35^ together with the SARC/J^36,37^ auxiliary basis set. Solvent effects (acetonitrile, MeCN) were taken into account in the optimizations using the C-PCM Gaussian charge scheme^38,39^ continuum solvation model. Frequency calculations showed that the optimized structures correspond to minima (no imaginary frequency).

Electronic excited states were computed using the time-dependent version of DFT (TD-DFT) with the same functional, as B3LYP has been shown to yield good results for computing absorption spectra of Ru-complexes.^40–45^ The Tamm–Dancoff approximation^46^ was used to compute the spectra with the scalar relativistic ZORA^47^ Hamiltonian, and the relativistically recontracted versions of the basis sets,^48^ ZORA-def2-TZVP and ZORA-TZVP on Ru. 150 singlet excited states were calculated for Ru(dpp) and 200 states for Ru(dppip-NO_2_). The stick spectra of the equilibrium geometries were convoluted with Gaussian functions employing a full width at half maximum (fwhm) of 0.35 eV to obtain their absorption spectra. Furthermore, 500 geometries for each Ru-complex were sampled^49^ from a temperature-dependent Wigner distribution^20,50^ at 0 K and 300 K excluding low-frequency vibrational modes below 40 cm^−1^ (50 cm^−1^ for 0H-Ru(dppip-NO_2_)). Out of these ensembles, subsets of 50 geometries were used to calculate the Wigner spectra using Gaussian functions with a fwhm of 0.30 eV. All DFT and TD-DFT calculations were performed with the ORCA 4.2 program package.^51,52^

The character of the electronic excited states was determined using an automatized charge transfer (CT) analysis of the transition density matrix performed with the program package TheoDORE,^53–55^ as described in Section S1 of the ESI.† For this, the Ru-complexes were divided into chromophoric fragments, which are depicted in Fig. S1 in the ESI.† The excited states are labeled as locally excited ligand centered (LC) states and metal centered (MC) states or charge transfer excitations, *i.e.*, metal-to-ligand charge transfer (MLCT), ligand-to-ligand charge transfer (LLCT), or ligand-to-metal charge transfer (LMCT) excitations. In Section S2 of the ESI,† a comparison to CAM-B3LYP TD-DFT calculations (Fig. S2–S4, ESI†) and to B3LYP absorption spectra including spin–orbit coupling between singlet and triplet excited states (Fig. S5, ESI†) can be found.

## 3 Results and discussion

The three 0H,1H,2H-Ru(dppip-NO_2_) complexes show a very similar distorted octahedral coordination geometry of the ruthenium center, analogous to that encountered in Ru(dpp).^6,8^ However, there are differences in the structure of the equatorial dppip-NO_2_ ligand. Specifically, we found an increase in the nitrophenyl dihedral angle (denoted by *φ*_ph–NO_2__, *cf.*Fig. 1b) with respect to the im-dpp ring system with increasing number of protons in the order of Ru-0H < Ru-1H < Ru-2H,^8^ see Table 1. That is, the nitrophenyl group lies basically in the plane of the aromatic im-dpp rings in the deprotonated Ru-0H complex, but it is rotated out of the im-dpp plane by a dihedral angle of up to −25° in the doubly protonated Ru-2H and slightly (−5°) in the singly protonated Ru-1H species, probably due to steric effects.^8^ The dihedral angle between the NO_2_ group and the phenyl ring of dppip-NO_2_, denoted by *φ*_NO_2__ in Fig. 1b, is zero in all three equilibrium structures, but plays an important role in the vibrational ensembles, as it will be described below.

**Table 1:** Ru(dppip-NO_2_) complexes with zero (0H), one (1H), and two (2H) protons on the imidazole group of dppip-NO_2_: nitrophenyl dihedral angle *φ*_ph–NO_2__ with respect to im-dpp ring system and HOMO−3–LUMO energy gap Δ*E*, adapted from ref. 8

Ru-complex	Number of H^⊕^	*φ*_ph–NO_2__	Δ*E* (L–H−3)
Ru-0H	0	−0.8°	3.1 eV
Ru-1H	1	−5°	3.4 eV
Ru-2H	2	−25°	3.8 eV

Alongside the structural changes with (de)protonation of the dppip-NO_2_ ligand, we found differences in the electronic structure as well. Notably, the energy gap between the HOMO−3 (highest occupied molecular orbital-3) and LUMO (lowest unoccupied molecular orbital) increases with increasing number of protons^8^ as summarized in Table 1. In the following, we shall investigate how these changes within the aromatic ring systems of dppip-NO_2_ upon (de)protonation influence the spectroscopic properties of Ru(dppip-NO_2_).

### 3.1 Absorption spectra of 0H,1H,2H-Ru(dppip-NO_2_)

**Fig. 2:**
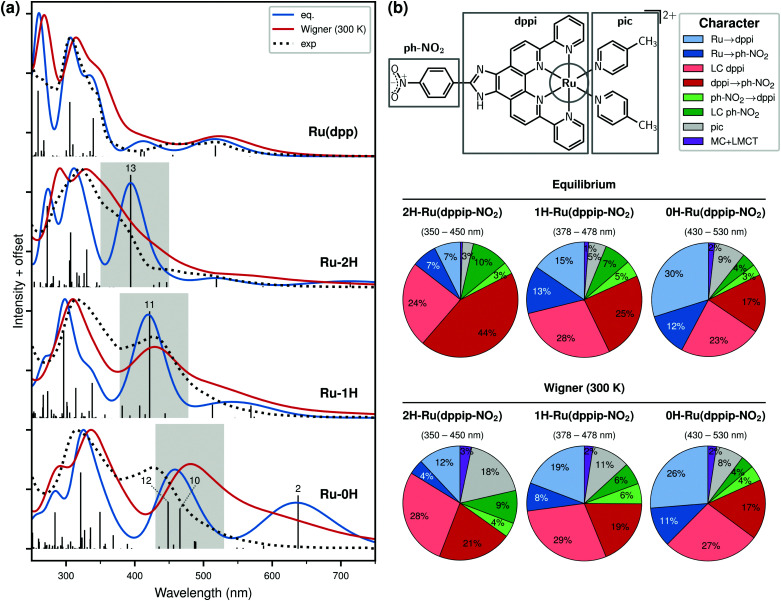
(a) Calculated absorption spectra of Ru(dpp) and 0H,1H,2H-Ru(dppip-NO_2_) using the equilibrium (eq.) structures (blue lines, adapted from ref. 8) and 300 K Wigner ensembles in MeCN (red lines). The computed spectra are compared to the experimental (exp.) spectra (dotted lines, ref. 8) of Ru(dpp) in MeCN and Ru(dppip-NO_2_) in Britton–Robinson buffer/MeCN at a pH of 1.95 (Ru-2H) and of 12.15 (Ru-1H, Ru-0H). (b) Corresponding *f*_osc_-weighted vis band characters of 0H,1H,2H-Ru(dppip-NO_2_) according to eqn (1) of the ESI;† the energy range of the vis band is shown above the pie charts. Colors indicate the character obtained using CT numbers between the specific molecular fragments shown at the top.

The computed absorption spectra of 0H,1H,2H-Ru(dppip-NO_2_) are shown in Fig. 2a. We shall first discuss the spectra obtained as vertical excitations carried out from the equilibrium geometries (blue lines), see also Section S3 of the ESI† for characters of the bands (Fig. S6–S9, ESI†) and natural transition orbitals (Fig. S10–S12, ESI†).

Compared to Ru(dpp), the 0H,1H,2H-Ru(dppip-NO_2_) complexes show a strong absorption band in the vis region (highlighted in gray) that is absent in Ru(dpp) and is responsible for their different absorption properties.^8^ Conspicuously, the peak maximum of this band in the convoluted spectra shifts to higher energies with increasing number of protons in the order of Ru-2H (394 nm) < Ru-1H (419 nm) < Ru-0H (458 nm).^8^ This band is due to transitions to the LUMO orbital, which is localized on the nitrophenyl group. Fig. 2b summarizes the contributions of the different transitions present within this vis band as pie charts. For this, all states within the energy range of the vis band (highlighted in gray in Fig. 2a) were considered and weighted by their respective oscillator strength according to eqn (1) of the ESI.† As can be seen, excitations to the equatorial dppip-NO_2_ ligand dominate the vis band.

**Fig. 3:**
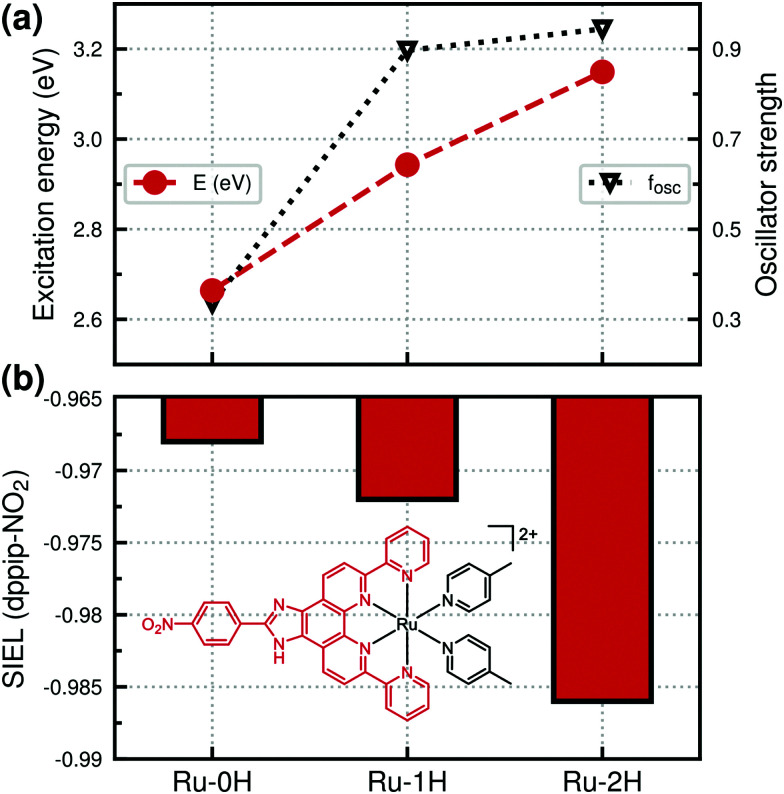
(a) Excitation energies and oscillator strengths *f*_osc_ of bright vis states S_10_, S_11_, S_13_ in 0H,1H,2H-Ru(dppip-NO_2_). (b) SIEL descriptors calculated for equatorial dppip-NO_2_ ligand for the bright vis states.

While mainly one bright state is responsible for the pronounced vis absorption band in Ru-2H and Ru-1H (states S_13_ and S_11_, respectively), two states (S_10_ and S_12_) with large oscillator strength underlie the vis band of Ru-0H. The excitation energies and oscillator strengths of the bright states, *i.e.*, S_13_ for Ru-2H, S_11_ for Ru-1H, and S_10_ for Ru-0H, are depicted in Fig. 3a. The S_12_ of Ru-0H is not considered as this is a state of different nature (mostly of Ru → dpp MLCT character, see the natural transition orbitals in Fig. S10 in the ESI†) that does not occur the Ru-2H,1H forms. Fig. 3a illustrates the clear blue shift of the excitations and the concomitant increase in oscillator strength of the S_10_, S_11_, S_13_ states in Ru-0H,1H,2H, respectively. However, the character of the involved states stays similar in all complexes, irrespective of the protonation state (see Fig. 2b). The natural transition orbitals of the S_10_, S_11_, and S_13_ states can be found in Fig. S11 in the ESI.† As previously reported by us,^8^ the transitions can be described as 
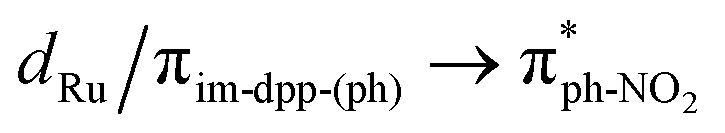
 excitations (mostly HOMO−3 → LUMO), where the *d*_Ru_ contribution decreases and the contribution of the phenyl ring (ph) increases with increasing number of protons. The MLCT character of the states therefore decreases slightly from Ru-0H to Ru-2H, which is accompanied by an increase of dppi → nitrophenyl LLCT and dppip-NO_2_-centered LC contributions. It is interesting to note that the energy shift of the S_10_, S_11_, and S_13_ states can be correlated with an increase in the HOMO−3–LUMO gap (*cf.*Table 1), which can be understood from the HOMO−3 → LUMO character of the transitions.^8^

How much a functionalized ligand attracts or repels an excited electron can be measured with the so-called substituent-induced exciton localization (SIEL) descriptor, introduced in ref. 56. If a particular group attracts the excited electron, SIEL is negative; if it repels it, SIEL is positive. From the SIEL values depicted in Fig. 3b, the protonated imidazole group of Ru-2H increases the positive charge on the acceptor ligand dppip-NO_2_, which further increases its attractive character (SIEL number close to −1). At the other extreme, in Ru-0H, the SIEL descriptor becomes less negative, which demonstrates the reduced attraction of the excited electron by the deprotonated dppip-NO_2_ ligand.

Ru-0H features another low-lying intense state, the S_2_ at 637 nm with a high oscillator strength of 0.45. It is characterized by a 
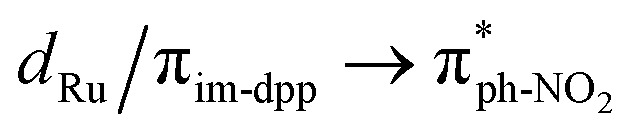
 LLCT/MLCT excitation (predominantly of HOMO → LUMO character), see the natural transition orbitals in Fig. S10 in the ESI.† It should be noted, though, that this state S_2_ has a high CT character (0.81, as estimated by TheoDORE^53^), so the B3LYP excitation energy may be too low. Yet, such an intense absorption at low energies is not observed in the Ru-1H,2H complexes, which exhibit rather weak *d*_Ru_
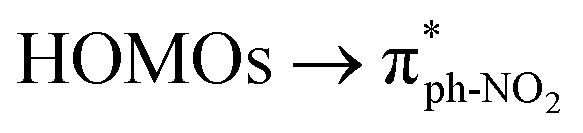
 LUMO MLCT transitions in this energy range (*f*_osc_ ≈ 0.05–0.1).

### 3.2 Effect of vibrational sampling

Vibrational effects may affect the band shapes in the absorption spectra and can lead to shifts of the absorption maxima.^14,15^ Additionally, it has been shown that vibrational motion can dramatically reduce the CT character of the absorption bands in nitro-aromatic compounds.^18–20^ Such effects are accounted for here by extending our analysis from the single frozen equilibrium geometry to 50 geometries for each Ru-complex, sampled from a Wigner distribution at 300 K. The corresponding calculated spectra are shown as red lines in Fig. 2a.

On first sight, it can be seen that the pH-dependent vis band of the Ru(dppip-NO_2_)-complexes is indeed sensitive to vibrational sampling compared to the spectra obtained from the equilibrium geometries, both in terms of intensity and position of the peak maximum. The intensity of the vis band decreases upon inclusion of nuclear motion. Further, vibrational sampling leads to a band broadening and most band maxima shift to lower energies by about 0.1 eV (up to 0.2 eV), in agreement with other literature examples.^14,15^ While it can be argued that such energy shifts lie within the error bar of TD-DFT,^21,57^ it is also fair to acknowledge that they do not affect systematically all chromophores or parts of the spectrum in the same manner. Therefore, it is imperative to consider vibrational effects on absorption spectra before discussing energy differences that are of the same order of magnitude due to methodological choices.

The peak maxima of the main absorption bands in the convoluted spectra of Ru(dpp) and 0H,1H,2H-Ru(dppip-NO_2_) are reported in Table 2. Distinct low-energy peaks are only observed as a tail of the band. In contrast, the spectrum of Ru(dpp) or other regions of the spectra of Ru(dppip-NO_2_) are only affected to a small extent. It seems that vibrational sampling is less important for describing the absorption spectra of more rigid complexes, such as Ru(dpp). In Ru(dppip-NO_2_), however, the changes are larger and we observe a better agreement with the experimental pH-dependent spectra,^8^ in particular for the Ru-1H,2H complexes, than that obtained from the single equilibrium geometry spectra. These two species, Ru-2H and Ru-1H, are speculated to be the main species in the experimental Ru(dppip-NO_2_) spectra in the investigated pH range (up to a pH of about 12).^8^ From the changes in the experimental pH-dependent spectra, which showed a single isosbestic point, a p*K*_a_ value of about 6.8 could be determined.^8^ It should be noted that higher pH values could not be reached in the titration experiments because a phase separation between MeCN and the buffer solution occurred upon further addition of NaOH and the Ru(dppip-NO_2_) complex began to partially precipitate,^8^ so significant amounts of the fully deprotonated Ru-0H species are probably not observed. Still, the Ru-0H species may also contribute to the absorption in the low-energy region of the experimental spectra in the upper end of the investigated pH range. Even more striking than the energy shift in the Wigner spectra is the drop in the intensity of the vis peak, in particular for the Ru-1H,2H species.

**Table 2:** Wavelength *λ*_max_ of prominent absorption band maxima in the convoluted spectra of Ru(dpp) and 0H,1H,2H-Ru(dppip-NO_2_) computed from the equilibrium geometries (eq.) and Wigner ensembles (0 K, 300 K); sh = peak shoulder. Changes in amount of CT to nitrophenyl group in 0H,1H,2H-Ru(dppip-NO_2_) spectra: bright states in the vis band, average character of vis band (*cf.* eqn (1) in the ESI) in spectra calculated from the equilibrium geometries (eq.) and the 300 K Wigner ensembles. UV-1,2 and vis-1,2 denote the different peak maxima in the UV range and visible range of the spectra, respectively

		*λ*_max_ absorption peak maxima (nm)		
		eq.	0 K Wigner	300 K Wigner
Ru(dpp)	UV-1	307	314	314
	UV-2	261	267	268
Ru-0H	vis-1	635		(sh)
	vis-2	458		481
	UV-1	325		336
	UV-2	285		292
Ru-1H	vis-1	419	419	429
	UV-1	298	310	310
Ru-2H	vis-1	394		(sh)
	UV-1	311		329
	UV-2	274		291
	
		CT character to nitrophenyl in vis band		
		State	eq.	300 K Wigner
Ru-0H	vis-2	S_10_ : 52%	30%	29%
Ru-1H	vis-1	S_11_ : 47%	39%	28%
Ru-2H	vis-1	S_13_ : 56%	52%	27%

Differently from what was found in nitrobenzene,^18^ at first sight the overall characters of the transitions of 0H,1H,2H-Ru(dppip-NO_2_) are little affected by vibrational sampling with few exceptions, see Fig. 2b and Fig. S7–S9 in the ESI.† In the Wigner spectra, the contribution of the axial pic ligands to the vis band increases compared to the equilibrium spectra (gray areas in the pie chart of Fig. 2b). In Ru-2H, the UV band/vis shoulder becomes less pure, *i.e.*, the dppi → nitrophenyl character decreases from 44% in the equilibrium spectra to 21% in the Wigner spectra. However, and most importantly, transitions involving the nitrophenyl group remain predominant in the vis region: Ru → dppip-NO_2_ MLCT transitions (blue) and dppi → dppip-NO_2_ LC/LLCT transitions (red) still dominate the vis bands, or vis shoulder in the case of Ru-2H, in the Wigner spectra.

A detailed analysis focusing on excitations to the nitrophenyl group shows the effect of the vibrational sampling more clearly. Table 2 summarizes the changes in the CT to the nitrophenyl group that occur in the vis band between the spectra of the equilibrium geometries and of the 300 K Wigner ensembles. The amount of CT to the nitrophenyl group, determined with TheoDORE, is reported for the bright vis states S_10_, S_11_, S_13_ of 0H,1H,2H-Ru(dppip-NO_2_) and compared to *f*_osc_-weighted averages over the vis absorption bands (as in eqn (1) in the ESI†) in the equilibrium spectra and the Wigner spectra. It should be recalled that not only one, but two states of different character, S_10_ and S_12_, are responsible for the vis absorption band in the Ru-0H equilibrium spectrum. Therefore, it exhibits a lower CT to the nitrophenyl group when considering both states underlying the absorption band compared to just the S_10_ state. Table 2 reveals that the CT to the nitrophenyl group decreases from about 40–50% in the equilibrium spectra to *ca.* 30% in the Wigner spectra.

### 3.3 Effect of temperature

**Fig. 4:**
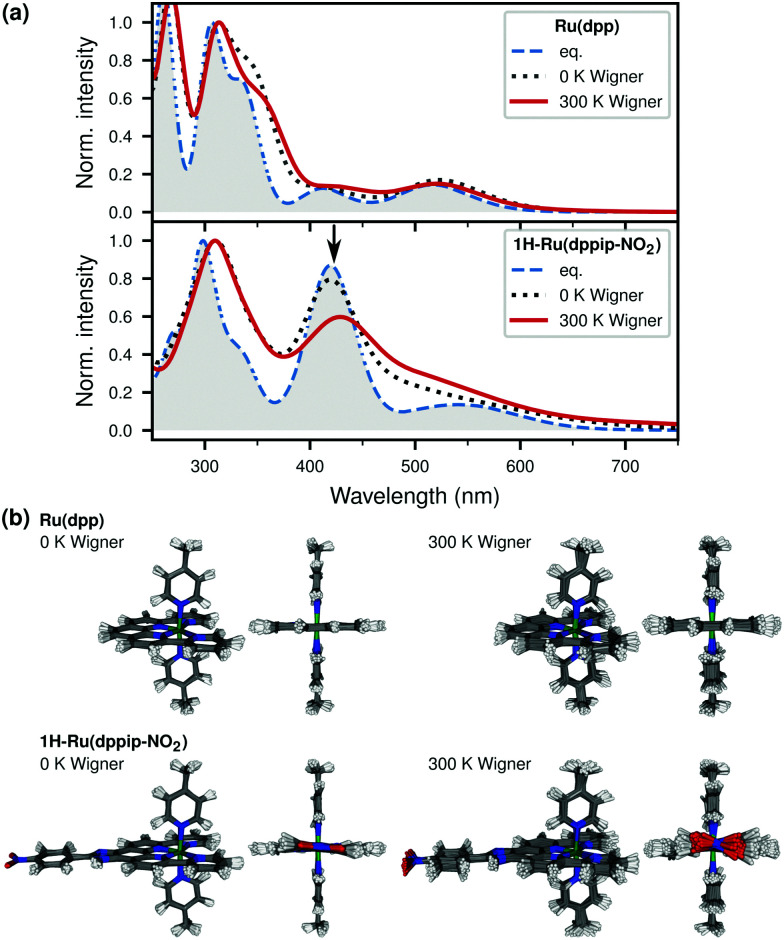
(a) Comparison of spectra of Ru(dpp) and 1H-Ru(dppip-NO_2_) computed from equilibrium geometries (eq.) and Wigner ensembles at 0 K and 300 K (50 geometries). (b) Superimposed structures of 0 K and 300 K Wigner ensembles.

Temperature effects on the absorption spectra were estimated by computing the spectra of Ru(dpp) and 1H-Ru(dppip-NO_2_) – the species which is important in the catalytically relevant pH range^8^ and the neutral form of the dppip-NO_2_ ligand – from ensembles sampled from Wigner distributions at 0 K and 300 K, see Fig. 4a. The flexibility achieved by increasing the temperature from 0 K to 300 K can be appreciated in the superpositions of structures shown in Fig. 4b. This flexibility is responsible for the differences obtained in the spectra at different temperatures – an effect that is clearly more visible in 1H-Ru(dppip-NO_2_).

The position of the main absorption peak maxima in the spectra of Ru(dpp) and 1H-Ru(dppip-NO_2_) computed from the equilibrium geometries and Wigner ensembles was reported in Table 2. Interestingly, while the general peak broadening and the red shift of the UV absorption band compared to the equilibrium spectra is already observed in the 0 K Wigner spectra, the decrease in intensity of the vis peak and its spreading over a broader energy range mainly occurs in the 300 K Wigner spectra around 420 nm (*cf.* 0 K and 300 K Wigner spectra of 1H-Ru(dppip-NO_2_) in Fig. 4a). Table 2 clearly demonstrates the shift of the vis absorption peak maximum of 1H-Ru(dppip-NO_2_) in the 300 K Wigner spectrum, whereas no such effect is observed in the spectrum computed from the 0 K ensemble. The red shift of the UV band in the 0 K spectrum is, however, not further increased in the 300 K ensemble, neither in 1H-Ru(dppip-NO_2_) nor in Ru(dpp). Due to its rigidity, temperature effects are less pronounced for Ru(dpp).

**Fig. 5:**
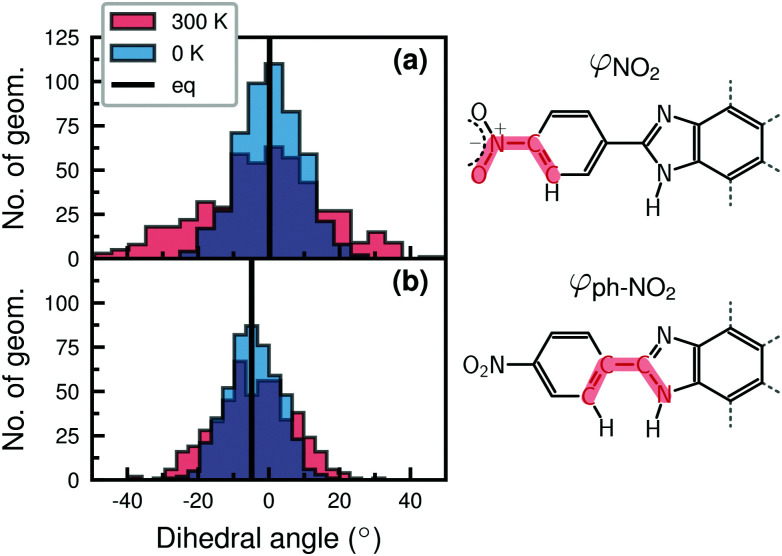
Distributions of dihedral angles in 1H-Ru(dppip-NO_2_) 0 K and 300 K Wigner ensembles (500 geometries) compared to eq. values: (a) NO_2_ dihedral angle *φ*_NO_2__, (b) nitrophenyl dihedral angle *φ*_ph–NO_2__.

In order to better understand the changes in the Wigner spectra of 1H-Ru(dppip-NO_2_), it is useful to analyze the structures of the vibrational ensembles. To this end, Fig. 5 compares the distributions of the NO_2_ dihedral angle *φ*_NO_2__ (Fig. 5a) and of the nitrophenyl dihedral angle *φ*_ph–NO_2__ with respect to the im-dpp ring (Fig. 5b) in the 1H-Ru(dppip-NO_2_) ensembles at 0 K and 300 K, respectively. First, we focus on structural changes in the Wigner ensembles compared to the equilibrium geometry. The main changes occur in the NO_2_ dihedral angle *φ*_NO_2__, which deviates from planarity in many structures, see histograms in Fig. 5a. This behavior is reminiscent to that found in another nitro-aromatic compound.^18^ Besides the NO_2_ group, the dihedral angle *φ*_ph–NO_2__ of the whole nitrophenyl group with respect to the im-dpp ring system is affected (Fig. 5b), and also the O–N–O angle shows changes. Other geometric parameters, such as the planarity of some of the aromatic rings, are also affected by the Wigner sampling, in contrast to the coordination sphere around ruthenium, which is hardly influenced.

Next, we compare the 0 K and 300 K Wigner ensembles of 1H-Ru(dppip-NO_2_). While in the 0 K ensemble only the vibrational ground states are populated, temperature can make excited vibrational states accessible.^14^ This is particularly relevant for low-frequency vibrational modes. However, care must be taken with low-frequency modes because geometries with overestimated atom displacements might occur in the Wigner ensemble. In addition, the Wigner ensemble could feature deformed geometries in the case of rotational motions, as such motions cannot be properly described in linear normal mode coordinates. This was, for example, the case with the nearly free imidazole torsion in [Re(CO)_3_(im)(phen)]^+^ (torsional mode at 7–10 cm^−1^, barrier of about 0.04 eV).^21^ Hence, in our analysis of 1H-Ru(dppip-NO_2_) we excluded low-frequency modes below 40 cm^−1^ – the nitrophenyl out-of-plane motion occurs at about 46 cm^−1^ – and checked for geometries with deformed ligands. No deformation of the dppip-NO_2_ ligand at higher NO_2_ or nitrophenyl dihedral angles are observed in the 1H-Ru(dppip-NO_2_) 300 K Wigner ensemble as shown in Fig. S13 in the ESI† (see also superimposed structures in Fig. 4b).

While both the nitrophenyl and the NO_2_ dihedral angles exhibit greater changes in the Wigner ensembles compared to the equilibrium values, a comparison of the 0 K and 300 K Wigner ensembles of 1H-Ru(dppip-NO_2_) (blue and red histograms in Fig. 5) reveals that the NO_2_ dihedral angle *φ*_NO_2__ is particularly affected by the inclusion of temperature effects in the Wigner sampling. That is, the distribution is still comparatively narrow around the equilibrium value of 0° in the 0 K ensemble, but larger deviations from planarity are observed at 300 K (Fig. 5a). In contrast, the distributions of the nitrophenyl dihedral angle *φ*_ph–NO_2__ (Fig. 5b) are similar in both the 0 K and the 300 K ensembles. Hence, no significant differences between the 0 K and the 300 K spectra are expected due to 1H-Ru(dppip-NO_2_) structures with larger nitrophenyl dihedral angles *φ*_ph–NO_2__ at 300 K. Therefore, it seems likely that the larger changes in the NO_2_ dihedral angle (*φ*_NO_2__) at higher temperatures are responsible for the changes in the vis peak between the 0 K and 300 K spectra of 1H-Ru(dppip-NO_2_). This will be inspected in more detail in the next section.

### 3.4 Effect of torsional motion

The motion of the NO_2_ group and, to a lesser extent, of the nitrophenyl group could rationalize the effect the Wigner sampling has on the vis band of 0H,1H,2H-Ru(dppip-NO_2_), as this band is largely characterized by excitations that involve the nitrophenyl group. Still, the Wigner spectra contain a convolution of changes of other geometrical parameters that might also influence the character, energy, and oscillator strength of some excitations. It should be recalled that besides the geometries in the 300 K Wigner ensembles, also the equilibrium structures of 0H,1H,2H-Ru(dppip-NO_2_) deviate in their nitrophenyl dihedral angle *φ*_ph–NO_2__, which increases with protonation of the imidazole group (*cf.*Table 1).

**Fig. 6:**
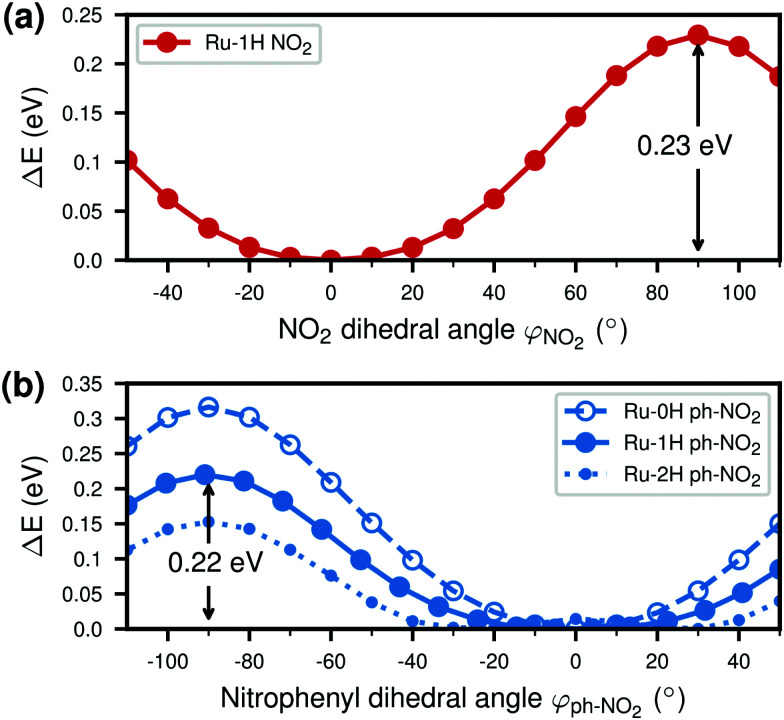
Relaxed dihedral angle scans: (a) NO_2_ dihedral angle *φ*_NO_2__ in 1H-Ru(dppip-NO_2_) and (b) nitrophenyl dihedral angle *φ*_ph–NO_2__ with respect to im-dpp ring system in 0H,1H,2H-Ru(dppip-NO_2_).

To investigate the effect of a torsional motion of the NO_2_ group or of the nitrophenyl ring more specifically, we performed relaxed scans of the corresponding dihedral angles in Ru-1H, see Fig. 6. The figure additionally presents a comparison to the relaxed *φ*_ph–NO_2__ scans of the Ru-0H,2H species (Fig. 6b). As can be seen, both the NO_2_ and the nitrophenyl torsion barriers are about 0.2 eV in Ru-1H. The nitrophenyl torsion barrier decreases with increasing number of protons from 0.32 eV in Ru-0H to 0.15 eV in Ru-2H. We then calculated absorption spectra for Ru-1H geometries in which the NO_2_ and nitrophenyl dihedral angles were varied incrementally from their equilibrium values (unrelaxed scans) to estimate the influence of this torsional motion on the absorption. See also Section S5 in the ESI† for absorption spectra and excited state characters computed using Ru-1H structures from the relaxed scans (Fig. S14, ESI†). Unrelaxed scans of all 0H,1H,2H-Ru(dppip-NO_2_) species can be found in Fig. S15 in the ESI,† which show the same trend and rather similar barriers as the relaxed scans. The corresponding absorption spectra based on these 0H,1H,2H-Ru(dppip-NO_2_) structures and excited state characters are shown in Fig. S16–S20 in the ESI.† As no significant differences were observed between the Ru-1H spectra or excited state characters computed from the relaxed (Fig. S14, ESI†) and unrelaxed scans, we focus in the following on the results from the unrelaxed scans.

**Fig. 7:**
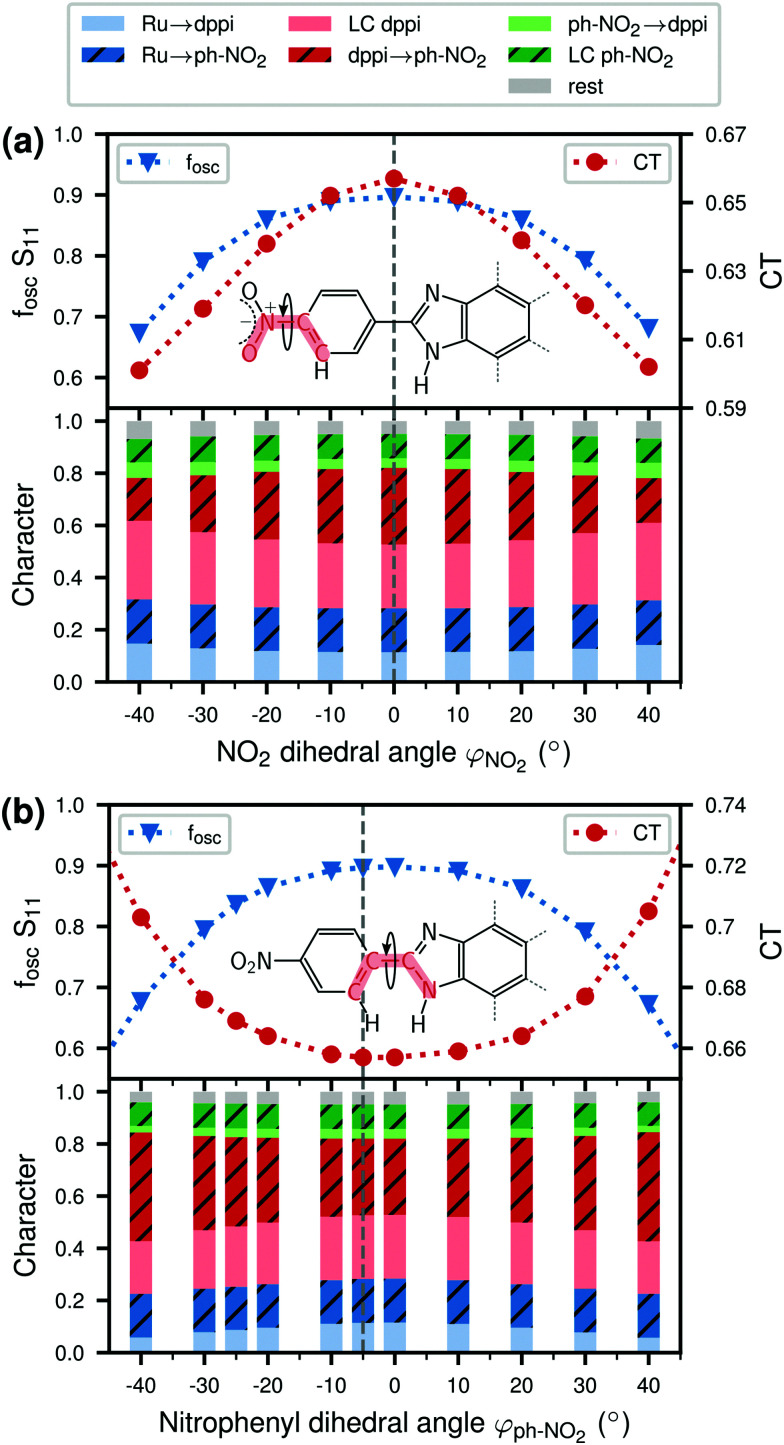
Effect of torsion on the oscillator strength *f*_osc_, the CT amount and the character of the bright vis state (S_11_) in 1H-Ru(dppip-NO_2_) structures along the (a) NO_2_*φ*_NO_2__ and (b) nitrophenyl *φ*_ph–NO_2__ dihedral angle. The dihedral angles were scanned from −40° to 40° (unrelaxed scans); the equilibrium angles are indicated by the dashed gray lines.

We observe significant effects of changes in the dihedral angles on the vis band of Ru-1H (see Fig. S17, ESI†). Both, the torsional motion of the NO_2_ group (Fig. S17a, ESI†) and of the whole nitrophenyl ring (Fig. S17b, ESI†) decrease the intensity of the vis absorption band. The influence of the NO_2_ and nitrophenyl dihedral angles on the bright vis state S_11_ of Ru-1H is illustrated in Fig. 7, which shows the changes in the oscillator strength *f*_osc_, the amount of CT, and the character of the S_11_ state as a function of the dihedral angles. Recall that the S_11_ state is the bright state dominating the vis absorption band of Ru-1H, based on the equilibrium spectrum (*cf.*Fig. 2a) or from the structures where the dihedral angles are more or less close to the equilibrium values.

As can be seen, the oscillator strength of S_11_ decreases significantly with increasing NO_2_ dihedral angle *φ*_NO_2__ (Fig. 7a) and different states become more important for the vis band at high NO_2_ dihedral angles |*φ*_NO_2__| ≥ 50°. Such high NO_2_ dihedral angles are, however, not observed in the Wigner ensemble underlying the absorption spectrum (*cf.*Fig. 5a) and are hence not relevant. This is why only the range between *φ*_NO_2__ = ±40° is shown in Fig. 7.

The CT character decreases with greater deviations of the NO_2_ dihedral angle *φ*_NO_2__ from planarity, which is mostly due to a decrease of the dppi → nitrophenyl LLCT character. In contrast to this decrease, the nitrophenyl dihedral angle *φ*_ph–NO_2__ scan shows an opposite effect. As seen in Fig. 7b, at large *φ*_ph–NO_2__ dihedral angles, where the nitrophenyl group is rotated out of the im-dpp plane, the largest contribution to the S_11_ state is the dppi → nitrophenyl LLCT transition (see also Fig. S18 in the ESI†). For nitrophenyl dihedral angles *φ*_ph–NO_2__ close to zero, on the other hand, the excitation seems to be more distributed over the equatorial dppip-NO_2_ ligand (dppi → dppip-NO_2_ LLCT/LC excitation) rather than a CT excitation from dppi to nitrophenyl. This means that a rotation of the nitrophenyl ring leads to an increasing localization of the excited electron on the nitrophenyl group and an increase in the CT character of the vis state (*cf.* Fig. S18, ESI†), with a concomitant decrease of the oscillator strength.

Like Ru-1H, the (de)protonated Ru-0H,2H forms of the catalyst also show a decrease in intensity and increase in the CT character of the vis states with increasing nitrophenyl dihedral angle *φ*_ph–NO_2__ (*cf.* Fig. S16, S19, and S20 in the ESI†). In Ru-0H, not only the vis absorption band, but also the low-energy peak around 635 nm is affected by the nitrophenyl torsion (Fig. S16, ESI†). The character of its underlying state (S_2_ in the equilibrium spectrum, recall Fig. 2a), 
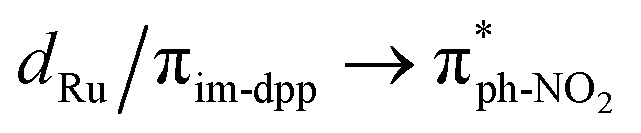
 (Fig. S10a, ESI†), is similar to the likewise torsion-sensitive bright vis state S_10_ of Ru-0H. That is, both states correspond to excitations to the nitrophenyl group and are obviously affected by torsional motions of or within the nitrophenyl ring. This could explain why no distinct peak around 635 nm is observed in the 300 K Wigner spectra of Ru-0H, but rather a continuous decrease in the absorption is seen in Fig. 2a.

The generally observed decrease in oscillator strength of the bright vis states S_10_, S_11_, and S_13_ in the 0H,1H,2H-Ru(dppip-NO_2_) equilibrium spectra with increasing nitrophenyl dihedral angle *φ*_ph–NO_2__, as well as in Ru-1H structures with greater deviations of the NO_2_ dihedral angle *φ*_NO_2__ from planarity, suggests that these states become less important for the vis absorption band in the spectra computed from the vibrational ensembles. This can explain the decrease in intensity of the vis absorption band and might also contribute to the decrease in the CT to the nitrophenyl group observed in the Wigner spectra.

**Fig. 8:**
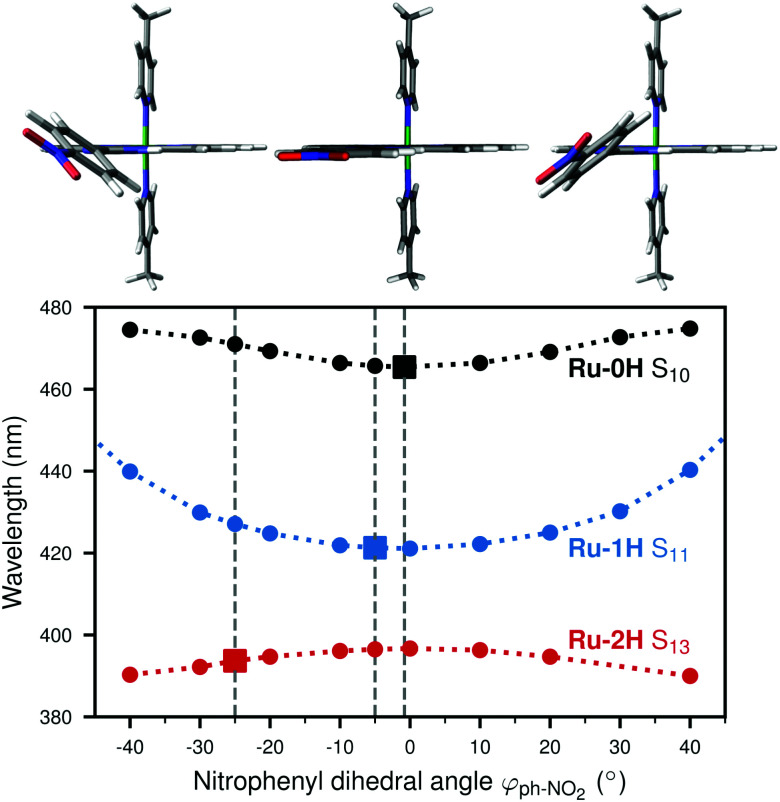
Changes in bright vis state (S_10_, S_11_, S_13_) wavelengths *vs.* nitrophenyl dihedral angle *φ*_ph–NO_2__ (unrelaxed scan) in 0H,1H,2H-Ru(dppip-NO_2_); 1H-Ru(dppip-NO_2_) structures with a *φ*_ph–NO_2__ of −40°, 0°, and 40° are shown at the top. The eq. angles are indicated by dashed gray lines.

Finally, we find it interesting to investigate whether the increasing nitrophenyl dihedral angle *φ*_ph–NO_2__ in the 0H,1H,2H-Ru(dppip-NO_2_) equilibrium structures is related to the blue shift of the vis band with increasing number of protons. To this end, Fig. 8 plots the wavelengths of the most important states (S_10_, S_11_, S_13_) of each complex as a function of the nitrophenyl dihedral angle. It can be seen that the increase in excitation energy with the number of protons does not correlate with an increase in the nitrophenyl dihedral angle from Ru-0H to Ru-2H. On the contrary, higher dihedral angles shift the vis states in Ru-0H and Ru-1H to lower energies, *i.e.*, further away from the Ru-2H vis state at 394 nm. Instead, as explained in ref. 8, we found that the increase in the HOMO−3–LUMO gap due to a stabilization of the HOMO−3 with increasing positive charge on the dppip-NO_2_ ligand is probably responsible for the shift of the bright vis states S_10_, S_11_, S_13_ to higher energies. A similar effect of (de)protonation was reported in a ruthenium 2,2'-biimidazole complex.^58^

## 4 Conclusions

The absorption spectrum of the Ru(II) water oxidation catalyst Ru(dppip-NO_2_) was analyzed in detail, paying particular attention to the influence of vibrational effects and torsional motions within its functionalized ligand dppip-NO_2_, which features an electron-withdrawing nitrophenyl group. Electronic excitations to this acceptor ligand result in a strong absorption of Ru(dppip-NO_2_) in the vis energy range, mainly due to one bright excited state, in contrast to its parent compound Ru(dpp). This absorption can be tuned by (de)protonation of the amphoteric imidazole moiety on dppip-NO_2_, which we studied by considering the different protonation forms of Ru(dppip-NO_2_) with zero (0H), one (1H), and two protons (2H). We found that the attractive character of dppip-NO_2_ for the excited electron (as quantified by the SIEL descriptor) increases with the number of protons on the imidazole group. Simultaneously, a blue shift and increase in oscillator strength of the bright vis states is observed in the different 0H,1H,2H-Ru(dppip-NO_2_) species.

This work highlights the importance of considering vibrational sampling to properly describe the pH-dependent vis band of Ru(dppip-NO_2_). We compared the absorption spectra computed from a single geometry (the minimum-energy equilibrium geometry, as done extensively in the literature), with spectra computed from a vibrational ensemble using the Wigner sampling approach. We showed that in order to achieve a good agreement with experimental pH-dependent spectra of Ru(dppip-NO_2_),^8^ it is necessary to go beyond the simple description based on vertical excitations from the equilibrium geometries by taking into account the vibrational motion of the nuclei.

Torsional motions within the functionalized ligand and vibrational effects have a significant influence on the vis absorption band of 0H,1H,2H-Ru(dppip-NO_2_), which decreases in intensity and broadens in energy. Yet, the overall band character stays similar in both the spectra computed from the equilibrium geometries and the vibrational ensembles: it is dominated by Ru → dppip-NO_2_ MLCT and dppi → dppip-NO_2_ LC/LLCT excitations.

Further, we showed that the simulation of absorption spectra should also consider the effect of the temperature employed in the experiments. While some peaks show a red shift and band broadening already in the 0 K Wigner ensemble, temperature has a prominent influence on the vis absorption band. Most notably, geometries with greater deviations of the NO_2_ dihedral angle from planarity become accessible at 300 K. The effect of the 300 K Wigner sampling on the vis band may well be related to a movement of the NO_2_ group and, to a lesser extent, of the nitrophenyl ring as a whole, which are among the most prominent structural changes in the Wigner ensembles. This is supported by scans performed along the NO_2_ and nitrophenyl dihedral angles and their impact on the UV-vis spectra of 0H,1H,2H-Ru(dppip-NO_2_), which show that the dominant bright states underlying the vis absorption band are particularly affected by these torsional motions. Their oscillator strengths decrease significantly at greater deviations of the NO_2_ or nitrophenyl dihedral angles from the equilibrium values. The importance of these states for the vis absorption band is thus reduced and the intensity of the band is decreased. Also the excited state characters, in particular the dppi → nitrophenyl LLCT character, are influenced by changes in the dihedral angles of the NO_2_ or nitrophenyl groups. Importantly, the charge transfer character to the nitrophenyl group of the vis absorption band is reduced in the Wigner spectra compared to the equilibrium spectra. Such a decrease in charge transfer character has been observed in organic nitro-aromatic compounds,^18–20^ and is shown here to be relevant in transition metal complexes as well.

In conclusion, we highlighted the sensitivity of the vis absorption band of the water oxidation catalyst Ru(dppip-NO_2_) not only to the protonation state of the imidazole group but also to nuclear motion, achieved here by Wigner sampling. Such effects are expected to play a role in other transition metal complexes that contain flexible ligands or nitro-aromatic motifs.

## Conflicts of interest

There are no conflicts to declare.

## Supplementary Material

CP-023-D1CP02748D-s001
