# Supplementary material for: The importance of finite temperature and vibrational sampling in the absorption spectrum of a nitro-functionalized Ru(ii) water oxidation catalyst
Source: Phys Chem Chem Phys. 2021 Aug 4;23(32):17724–33. doi: 10.1039/d1cp02748d (PMC8371993; doi:10.1039/d1cp02748d)
Supplement: CP-023-D1CP02748D-s001 [file CP-023-D1CP02748D-s001.pdf]

Electronic Supplementary Material (ESI) for Physical Chemistry Chemical Physics.
This journal is © the Owner Societies 2021

Supporting Information: The importance of finite temperature and vibrational sampling in the absorption spectrum of a nitro-functionalized Ru(II) water oxidation catalyst

Anna M. Wernbacher* and Leticia González*

Institute of Theoretical Chemistry, Faculty of Chemistry, University of Vienna, 1090 Vienna, Währinger Straße 17, Austria.

E-mail: anna.wernbacher@univie.ac.at; leticia.gonzalez@univie.ac.at

Contents

S1 Excited state characterization with TheoDORE **S2**

S2 TD-DFT: Method comparison **S5**

S3 Absorption spectra of Ru(dppip-NO_2) and Ru(dpp) **S9**

S4 Wigner ensembles of 1H-Ru(dppip-NO_2) **S15**

S5 NO_2 and nitrophenyl dihedral angle scans **S16**

S5.1 Relaxed scans of 1H-Ru(dppip-NO_2) S16

S5.2 Unrelaxed scans S18

S5.2.1 0H-Ru(dppip-NO_2) S19

S5.2.2 1H-Ru(dppip-NO_2) S20

S5.2.3 2H-Ru(dppip-NO_2) S23

S5.2.4 Comparison of 0H,1H,2H-Ru(dppip-NO_2) S24

S6 Optimized geometries **S25**

References **S34**

S1 Excited state characterization with TheoDORE

The excited state characters were determined with the TheoDORE[S1–S3] program package, based on a charge transfer (CT) analysis of the one electron transition density matrix. This allows for a quantitative analysis of the excited states and, being automatized, is especially useful for analyzing the characters in the spectra computed from an ensemble of geometries like in the Wigner spectra. The starting point of the CT analysis is a partitioning of the one electron transition density matrix between the ground state and an excited state among predefined molecular fragments (A, B, . . .).[S2,S3] The Ru-complexes were divided into the

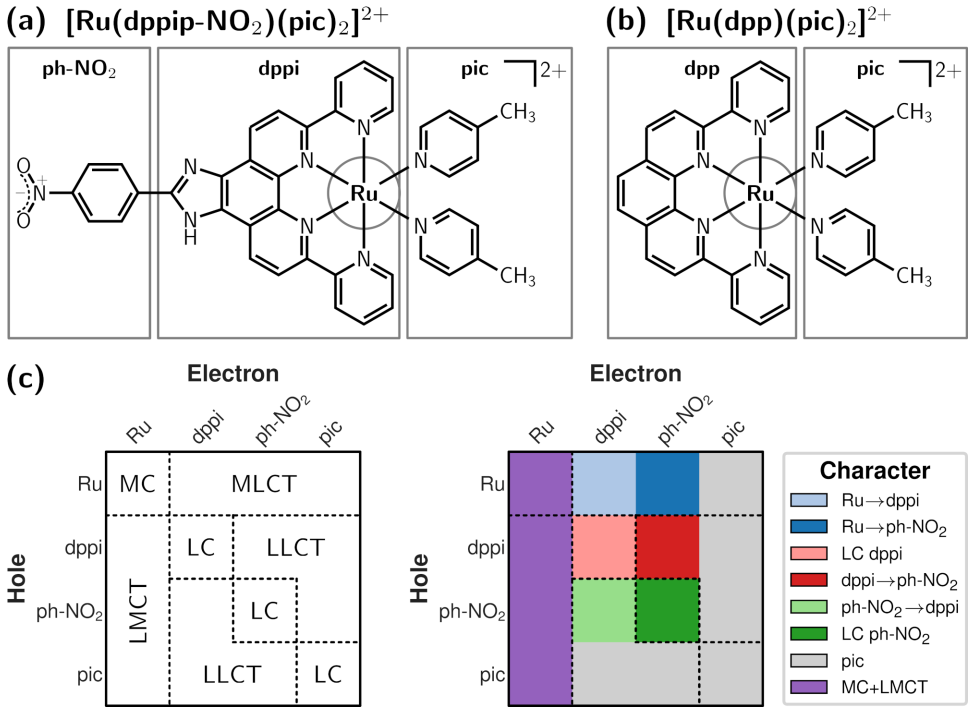

Figure S1: Fragmentation of Ru-complexes for CT analysis with TheoDORE. **(a)** Four fragments of Ru(dppip-NO_2): 1. Ru, 2. dppi, 3. pic, 4. nitrophenyl (ph-NO_2); **(b)** three fragments of Ru(dpp): 1. Ru, 2. dpp, 3. pic. **(c)** Matrix plot of CT numbers Ω_{AB} showing the excitation hole on the vertical axis and the excited electron on the horizontal axis.

fragments shown in Fig. S1a–b based on a hierarchical clustering[S4] ansatz; Ru(dppip-NO_2) is divided in four fragments (1 is the Ru atom, 2 the dppi fragment, 3 the nitrophenyl fragment and 4 the pic ligands) and Ru(dpp) in three (Ru, dpp ligand, and pic ligands). A Löwdin-type population analysis scheme was used to yield the CT numbers Ω_{AB} for a particular excited state, which can be visualized in the form of a matrix plot or so-called electron–hole correlation plot (Fig. S1c).[S2,S4] Here, the diagonal elements Ω_{AA} correspond to local excitations (on fragment A), whereas off-diagonal elements Ω_{AB} $A \neq B$ correspond to CT contributions to the excited state character.[S2,S4] Figure S1c shows an example plot of the 4×4 Ω-matrix of Ru(dppip-NO_2), where the excitation hole coordinate is plotted along the vertical axis and the excited electron coordinate along the horizontal axis. Different excited state characters are indicated in the figure: Ru $\rightarrow$ dppi and Ru $\rightarrow$ nitrophenyl metal-to-ligand charge transfer (MLCT) excitations are shown in blue, dppi $\rightarrow$ dppi ligand-centered (LC) and dppi $\rightarrow$ nitrophenyl ligand-to-ligand charge transfer (LLCT) excitations in red, nitrophenyl $\rightarrow$ dppi LLCT and nitrophenyl $\rightarrow$ nitrophenyl LC excitations in green, excitations from/to the axial pic ligands in gray, and metal-centered (MC) Ru d–d and ligands $\rightarrow$ Ru ligand-to-metal charge transfer (LMCT) excitations in violet. This color scheme is used below and in the main paper in the discussion of the absorption spectra of the Ru-complexes.

The relative character (char) of the vis absorption band was determined by summing over all states i within the respective energy range and weighting them by their oscillator strength (f_{osc}) according to

$$\text{char}_x = \frac{\sum_{i \in \text{vis band}}^{\text{states}} \text{char}_x(i) \cdot f_{\text{osc}}(i)}{\sum_{i \in \text{vis band}}^{\text{states}} f_{\text{osc}}(i)} \tag{1}$$

such that all characters x (x = Ru $\rightarrow$ dppi MLCT, Ru $\rightarrow$ nitrophenyl MLCT, dppi LC, dppi $\rightarrow$ nitrophenyl LLCT, nitrophenyl $\rightarrow$ dppi LLCT, nitrophenyl LC, transitions from/to pic, Ru MC and ligands $\rightarrow$ Ru LMCT) sum up to 100% ($\sum_x \text{char}_x = 1$).

S2 TD-DFT: Method comparison

For comparison to the B3LYP spectra presented in the main paper, we additionally report here TD-DFT calculations using the range-separated CAM-B3LYP[S5] functional. The B3LYP and CAM-B3LYP excitation energies are shown in Fig. S2, where the bright states are highlighted and labeled. We performed state overlap calculations with WFoverlap[S6] to compare the nature of the B3LYP and CAM-B3LYP excited states. The black lines connecting the bright states in Fig. S2 indicate the overlap between the respective excited states. It can be seen that CAM-B3LYP generally predicts higher excitation energies than B3LYP.

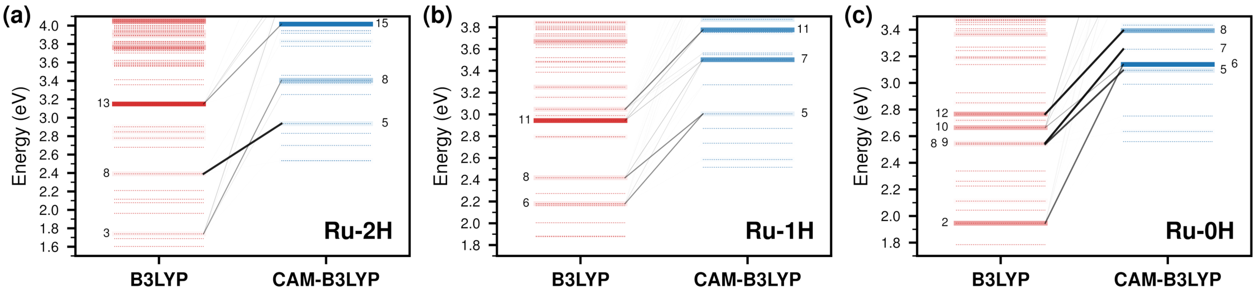

Figure S2: Comparison of B3LYP excitation energies with CAM-B3LYP results. States with a high oscillator strength are highlighted, while dark states are shown as dotted lines. The correlation of the bright B3LYP and CAM-B3LYP states is shown by the black lines, where the line width corresponds to the overlap of the excited states. **(a)** 2H-Ru(dppip-NO_2), **(b)** 1H-Ru(dppip-NO_2), **(c)** 0H-Ru(dppip-NO_2).

Thus, a blue shift of the CAM-B3LYP spectra is observed compared to the experiments, which can be seen in Fig. S3. The main band character, represented by the pie charts in Fig. S3 on the right, is similar to that of the B3LYP spectra: the intense band is dominated by Ru $\rightarrow$ dppi MLCT and dppi $\rightarrow$ dppip-NO_2 LC/LLCT excitations. CAM-B3LYP predicts a higher contribution of excitations to dppi, whereas the importance of excitations to ph-NO_2 decreases to some extent compared to the B3LYP equilibrium spectra. Besides the similar characters of the underlying excited states, both B3LYP and CAM-B3LYP predict a red shift of the absorption band with decreasing degree of protonation of the dppip-NO_2 ligand. This red shift also agrees with the experimental observation[S7] of a higher intensity at longer wavelengths with increasing pH. Due to the better agreement of the B3LYP spectra with

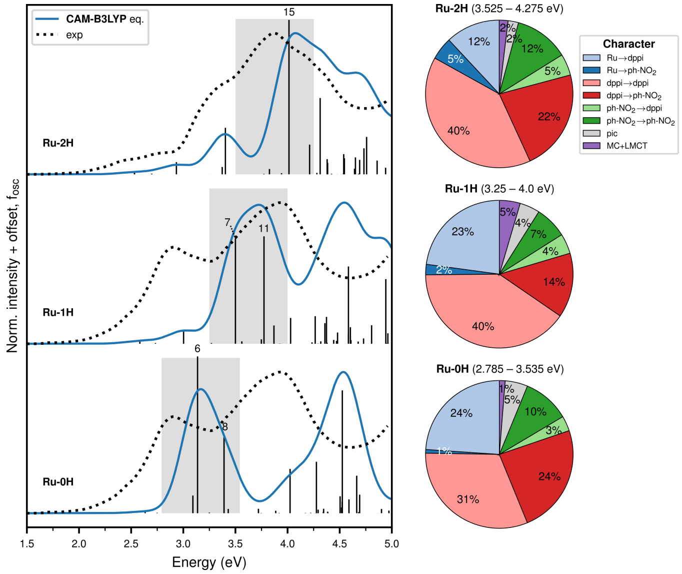

Figure S3: CAM-B3LYP absorption spectra of 0H,1H,2H-Ru(dppip-NO_2) based on the equilibrium (eq.) structures (blue lines). The computed spectra are compared to the experimental (exp.) spectra (dotted lines, adapted from Ref. S7) of Ru(dppip-NO_2) in Britton-Robinson buffer/MeCN at a pH of 1.95 (Ru-2H) and of 12.15 (Ru-1H, Ru-0H). The corresponding f_{osc}-weighted average band characters according to eqn (1) are shown on the right; the energy range of the band is shown above the pie charts.

the experiments, which was also observed previously for other Ru-complexes,[S8–S11] we chose B3LYP for the spectra in the main paper. In addition, for illustrative purposes, we compare here the experimental pH-dependent spectra (adapted from Ref. S7) with the B3LYP and CAM-B3LYP pH-dependent spectra in Fig. S4. For the latter, we considered the computed spectra of all three 2H,1H,0H-Ru(dppip-NO_2) species and scaled their intensities using the experimental $pK_{a,1}$ value of 6.8[S7] and an estimated $pK_{a,2}$ of 14 to demonstrate the influence of a third species on the pH-dependent spectra. Further details can be found in Ref. S7. Please note the different x axis scale for the CAM-B3LYP spectra in Fig. S4b. The figure illustrates that both B3LYP and CAM-B3LYP would predict a third rising band at high pH values in the low energy region if the 0H-Ru(dppip-NO_2) species contributed to the spectra to a greater extent. Since this is not observed in the experiments, this could indicate that large amounts of 0H-Ru(dppip-NO_2) do not occur in the experimentally investigated pH range.

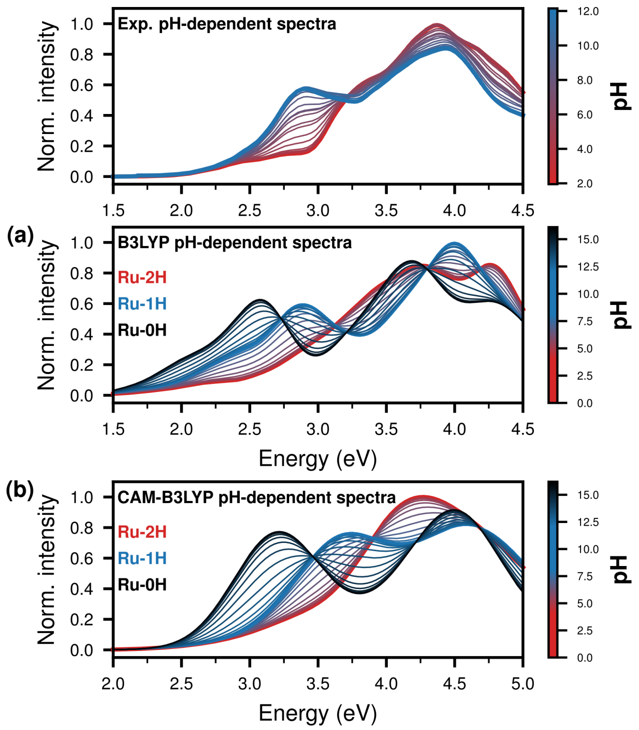

Figure S4: Ru(dppip-NO_2) pH-dependent absorption spectra: experimental spectra in Britton-Robinson buffer/MeCN (reproduced from Ref. S7) compared to **(a)** B3LYP 300 K Wigner spectra (adapted from Ref. S7) and **(b)** CAM-B3LYP eq. spectra (employing a large full width at half maximum of 0.7 eV to be better comparable to the Wigner spectra).

Furthermore, we investigated the role of spin–orbit coupling for the absorption spectra of the 2H,1H,0H-Ru(dppip-NO_2) complexes. Fig. S5 presents the B3LYP spectra without and with spin–orbit coupling between singlet and triplet states[S12] taken into account. As can be seen, no significant differences are observed in the spectra. Therefore, we have calculated only singlet excited states in the main paper and have not considered spin–orbit coupling further.

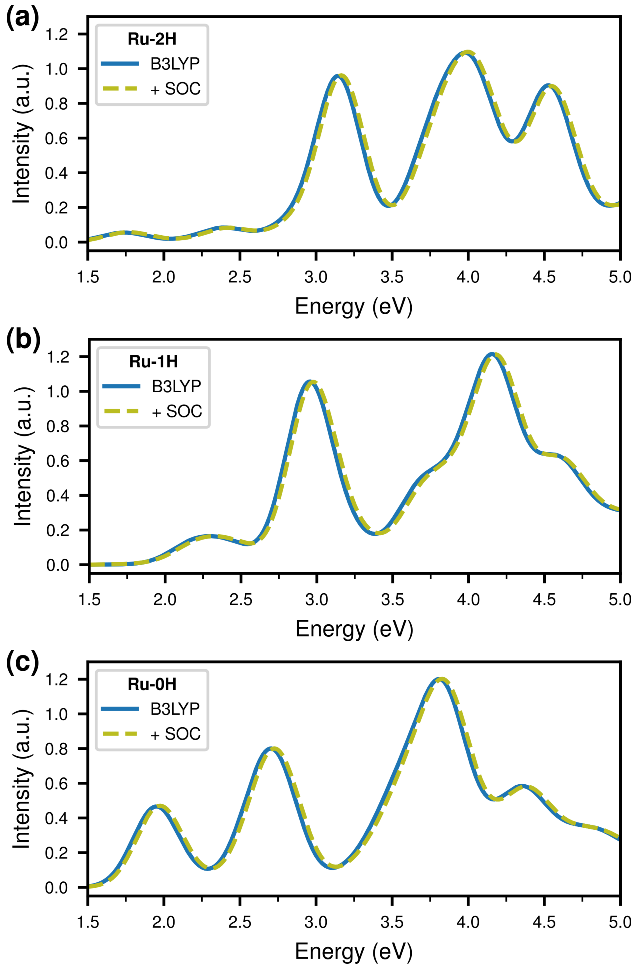

Figure S5: B3LYP absorption spectra without spin–orbit coupling (solid lines) and including spin–orbit coupling (dashed lines): **(a)** 2H-Ru(dppip-NO_2), **(b)** 1H-Ru(dppip-NO_2), **(c)** 0H-Ru(dppip-NO_2).

S3 Absorption spectra of Ru(dppip-NO_2) and Ru(dpp)

The equilibrium spectra of the Ru-complexes were reported by us previously in Ref. S7. Here, we focus on the nature of the absorption bands in the Wigner spectra compared to the equilibrium spectra and show natural transition orbitals of the most intense states of the equilibrium spectra for clarity. Fig. S6 (Ru(dpp)) and Figs. S7 – S9 (0H,1H,2H-Ru(dppip-NO_2)) present a comparison of the absorption spectra calculated using the equilibrium geometries of the Ru-complexes (at the top) with the spectra calculated from the respective 300 K Wigner ensembles (at the bottom). The spectra were decomposed into contributions from different excitation characters indicated by the color scheme. The equilibrium geometries and superimposed structures of the 300 K Wigner ensembles of Ru(dpp) and of 0H,1H,2H-Ru(dppip-NO_2) are presented in Figs. S6 – S9 on the right.

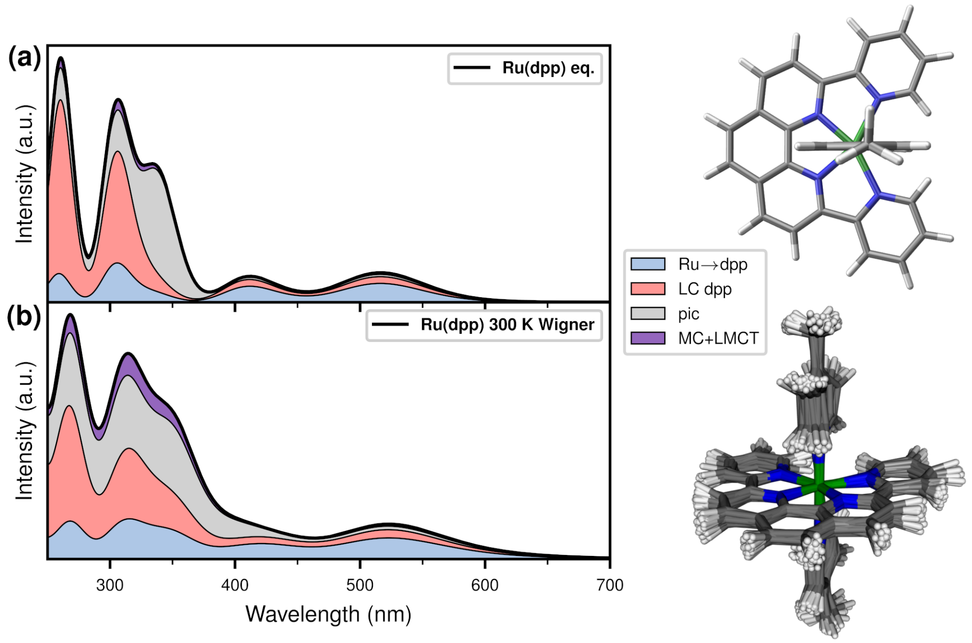

Figure S6: Absorption spectrum of Ru(dpp) and decomposition into different excitation characters: **(a)** equilibrium geometry, **(b)** 300 K Wigner ensemble (50 geometries).

First, we focus on the character of the low-energy bands in the spectra. MLCT excitations to the equatorial ligand are mainly important in the low-energy range of the spectra of Ru(dpp) and 1H,2H-Ru(dppip-NO_2), whereas the contribution of ligand-based LC and

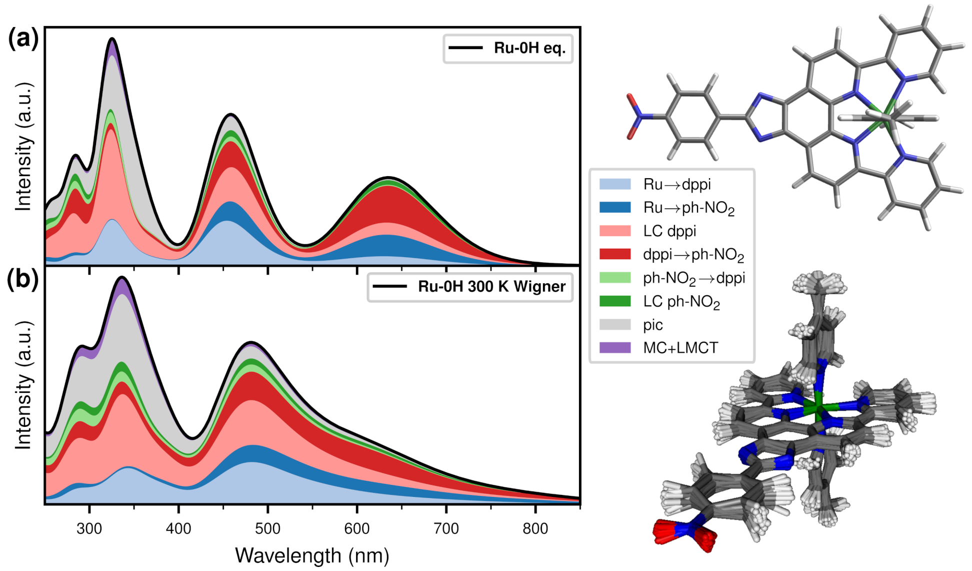

Figure S7: Absorption spectrum of 0H-Ru(dppip-NO_2) and decomposition into different excitation characters: **(a)** equilibrium geometry, **(b)** 300 K Wigner ensemble (50 geometries).

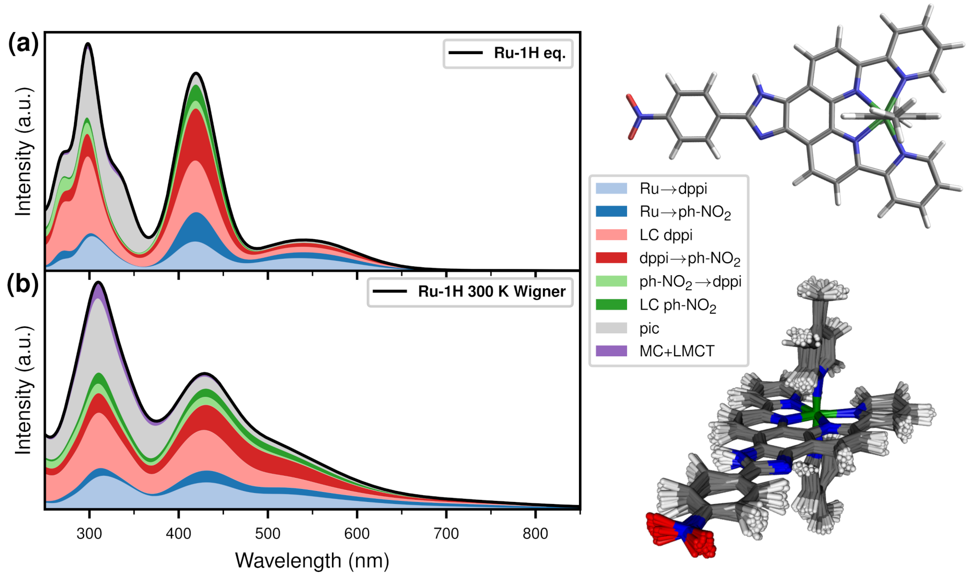

Figure S8: Absorption spectrum of 1H-Ru(dppip-NO_2) and decomposition into different excitation characters: **(a)** equilibrium geometry, **(b)** 300 K Wigner ensemble (50 geometries).

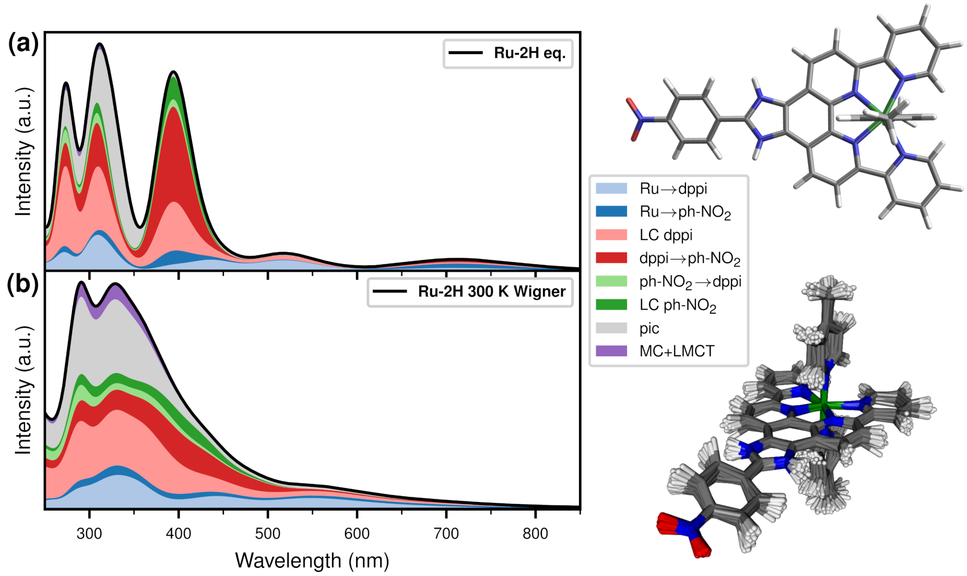

Figure S9: Absorption spectrum of 2H-Ru(dppip-NO_2) and decomposition into different excitation characters: **(a)** equilibrium geometry, **(b)** 300 K Wigner ensemble (50 geometries).

LLCT transitions increases at higher energies. In the 2H,1H,0H-Ru(dppip-NO_2) equilibrium spectra, MLCT excitations from d_{Ru}-based HOMOs to the $\pi^*_{\text{ph-NO}_2}$ LUMO and to higher-lying LUMO+1,2 of π^*_{dpp} character are observed at wavelengths greater than 500 nm. Similarly, Ru(dpp) exhibits MLCT excitations from the d_{Ru} HOMOs to the π^*_{dpp} LUMO and LUMO+1,2 above 400 nm. In contrast to the rather weak d_{Ru} HOMOs $\rightarrow$ $\pi^*_{\text{ph-NO}_2}$ LUMO MLCT transitions in 1H,2H-Ru(dppip-NO_2) (oscillator strengths $f_{\text{osc}} \approx 0.05 - 0.1$), 0H-Ru(dppip-$NO_2$) has a state with a high f_{osc} of 0.45 at low energies of mainly HOMO $\rightarrow$ LUMO character, the S_2 at 637 nm. The character of this S_2 state can be described as $d_{Ru}/\pi_{\text{im-dpp}} \rightarrow \pi^*_{\text{ph-NO}_2}$ LLCT/MLCT transition as shown by the natural transition orbitals depicted in Fig. S10. It should be noted that the HOMO in 0H-Ru(dppip-NO_2) corresponds to a $d_{Ru}/\pi_{\text{im-dpp}}$ orbital, whereas 1H,2H-Ru(dppip-NO_2) (and also Ru(dpp)) have nearly degenerate HOMOs of d_{Ru} character.[S7]

The most remarkable difference in the spectra of Ru(dpp) and 0H,1H,2H-Ru(dppip-NO_2) is the strong absorption band of the latter complexes in the visible (vis) energy range.[S7] The underlying bright states can be characterized as $d_{Ru}/\pi_{\text{im-dpp-ph}} \rightarrow \pi^*_{\text{ph-NO}_2}$ LLCT/MLCT excitations[S7] as shown by the natural transition orbitals in Fig. S11. Hence, excitations

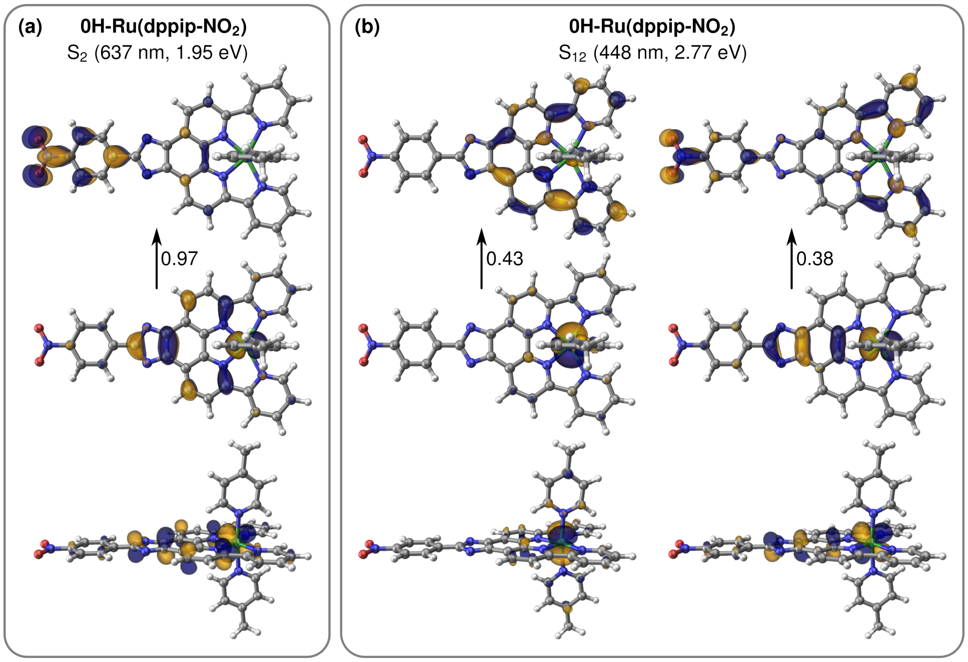

Figure S10: Natural transition orbitals of **(a)** the S_2 state and **(b)** the S_{12} state of 0H-Ru(dppip-NO_2). The two dominant contributions to the S_{12} state are shown.

to the electron-withdrawing nitrophenyl group seem to be the main reason for the different absorption properties of Ru(dpp) and 0H,1H,2H-Ru(dppip-NO_2) in the vis region.[S7] Another bright state, the S_{12} at 448 nm, of mostly of Ru $\rightarrow$ dpp MLCT character (see the natural transition orbitals in Fig. S10) contributes to the vis absorption band in 0H-Ru(dppip-NO_2).[S7]

An intense MLCT state that is observed in all complexes in the near UV range is the excitation from Ru to the axial pic ligands ($d_{\mathrm{Ru}} \rightarrow \pi^*_{\mathrm{pic}}$). This transition is not affected by the modification of the equatorial ligand, *i.e.*, it occurs at about the same energy with similar oscillator strengths f_{osc} in both the Ru(dpp) and 1H-Ru(dppip-NO_2) equilibrium spectra (at 339 nm with f_{osc} of 0.32 and at 338 nm with f_{osc} of 0.29 in Ru(dpp) and in 1H-Ru(dppip-NO_2), respectively, see Fig. S12). Also (de)protonation of the imidazole group has only small effects shifting the wavelength to 330 nm (f_{osc} of 0.32) in 2H-Ru(dppip-NO_2) and to 349 nm (f_{osc} of 0.31) in 0H-Ru(dppip-NO_2).

In the UV range, the spectra are dominated by intense transitions involving the equatorial

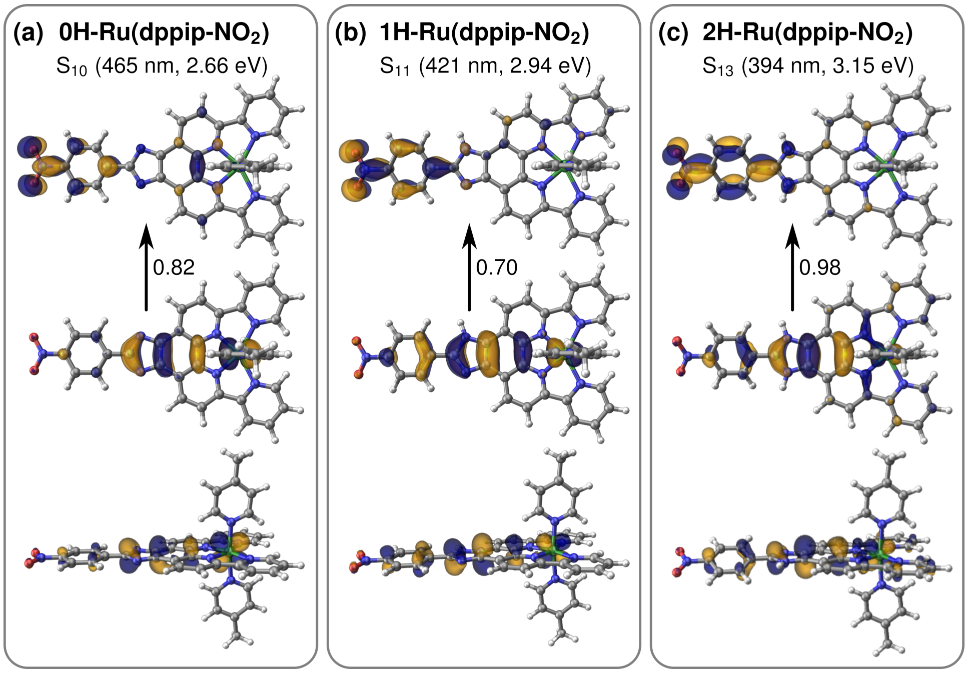

Figure S11: Natural transition orbitals of bright vis states **(a)** S_{10} in 0H-Ru(dppip-NO_2), **(b)** S_{11} in 1H-Ru(dppip-NO_2), and **(c)** S_{13} in 2H-Ru(dppip-NO_2); reproduced from Ref. S7.

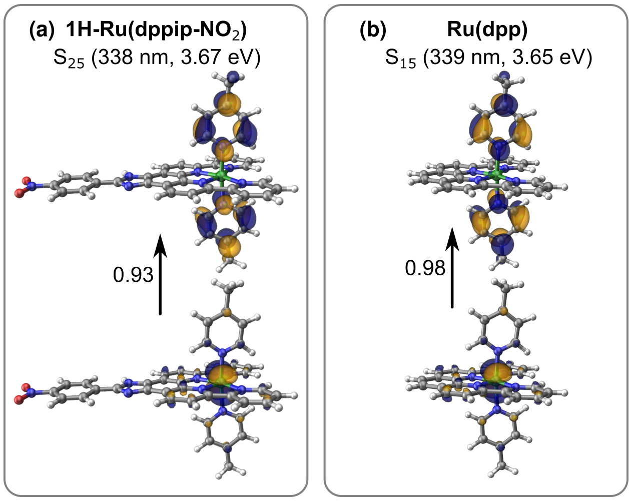

Figure S12: Natural transition orbitals of $d_{Ru} \rightarrow \pi^*_{pic}$ MLCT states **(a)** S_{25} in 1H-Ru(dppip-NO_2) and **(b)** S_{15} in Ru(dpp).

dpp or dppip-NO_2 ligand and excitations at the pic ligands, whose importance increases in the Wigner spectra compared to the equilibrium spectra.

These assignments agree with literature on Ru(dpp), where low-energy bands in the vis region were usually attributed to $d_{Ru} \rightarrow \pi^*_{dpp}$ MLCT transitions followed by MLCT transitions to the axial pic ligands at higher energies, and the intense UV band was suggested to be mainly caused by dpp-ligand-based $\pi \rightarrow \pi^*$ LC transitions.[S13,S14] Previous TD-DFT (B3LYP) calculations of Ru(dpp) in water found MLCT transitions at 519 nm,[S15] similar to the MLCT band at 517 nm calculated for the Ru(dpp) equilibrium geometry in acetonitrile (MeCN) in this work.

S4 Wigner ensembles of 1H-Ru(dppip-NO_2)

As mentioned in the main text, care must be taken when applying the finite temperature Wigner sampling to systems that have low-frequency modes or for torsional motions.[S16] We therefore excluded low-frequency modes below 40 cm^{-1} in the Wigner sampling. To check for a possible deformation of the dppip-NO_2 ligand, the distances between two opposite C atoms or H atoms in the nitrophenyl ring were analyzed as a function of the NO_2 and nitrophenyl dihedral angles in the Wigner ensemble; this is shown in Fig. S13 taking 1H-Ru(dppip-NO_2) as an example. The figure shows that no deformation of the nitrophenyl group of the dppip-NO_2 ligand is observed at higher deviations of the dihedral angles from their equilibrium values in the 300 K Wigner ensemble. This may also be seen in the superimposed structures of the 300 K Wigner ensembles of the Ru-complexes shown above in Figs. S6 – S9. That is, no exaggerated distances are seen here for 1H-Ru(dppip-NO_2) such as those reported for a 300 K Wigner ensemble of a Re-complex that exhibits a nearly free ligand torsion.[S16]

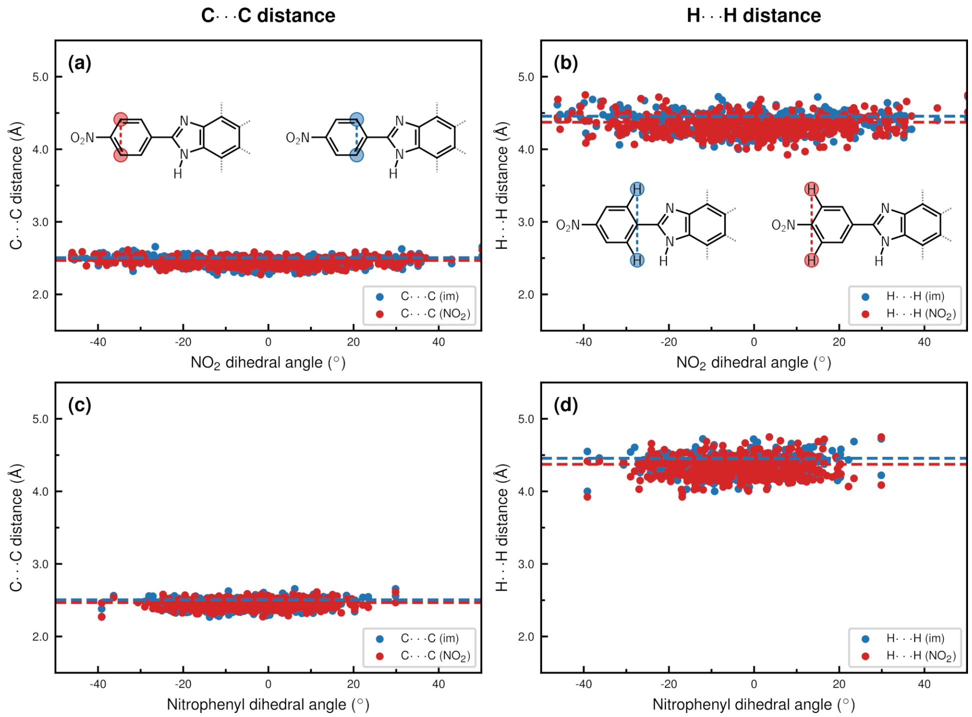

Figure S13: C–C and H–H distances in nitrophenyl group of dppip-NO_2 as a function of **(a)**–**(b)** the NO_2 and **(c)**–**(d)** the nitrophenyl dihedral angle in 1H-Ru(dppip-NO_2) 300 K Wigner ensemble (500 geometries). The equilibrium distances are shown as dashed lines.

S5 NO_2 and nitrophenyl dihedral angle scans

To determine the influence of torsional motions of or within the nitrophenyl group on the absorption spectra of 0H,1H,2H-Ru(dppip-NO_2) separately, scans of the NO_2 and nitrophenyl dihedral angles were performed and the spectra computed from these structures.

S5.1 Relaxed scans of 1H-Ru(dppip-NO_2)

In the main text, the absorption spectra and excited state characters obtained from unrelaxed NO_2 and nitrophenyl dihedral angle scans of 1H-Ru(dppip-NO_2) were shown and compared to the corresponding unrelaxed scans of the 0H,2H-Ru(dppip-NO_2) complexes. For comparison, the absorption spectra were additionally calculated from 1H-Ru(dppip-NO_2) structures from relaxed scans of the NO_2 and nitrophenyl dihedral angles, respectively, which are shown below in Fig. S14a–b. As in the unrelaxed scans, a significant decrease in intensity of the vis peak is observed. Fig. S14c–d presents the changes in the oscillator strength, CT number, and character of S_{11}, *i.e.*, of the bright vis state in the 1H-Ru(dppip-NO_2) equilibrium spectrum, with increasing deviations of the NO_2 dihedral angle (Fig. S14c) and nitrophenyl dihedral angle (Fig. S14d) from their equilibrium values. The same results are observed as before in the unrelaxed scans, *i.e.*, the oscillator strength of S_{11} drops quickly at higher dihedral angles. In the case of the NO_2 dihedral angle scan, this is accompanied by a *decrease* in the CT character, which is mainly due to a decrease of the dppi $\rightarrow$ nitrophenyl LLCT contribution shown in dark red in Fig. S14c. At high NO_2 dihedral angles $\varphi_{NO_2} \geq 50°$, states other than S_{11} become more important for the vis absorption band. Therefore, only the range between 0° and 40° is shown in Fig. S14c. The nitrophenyl dihedral angle scan (Fig. S14d) shows an opposite effect, *i.e.*, the CT character, especially the dppi $\rightarrow$ nitrophenyl LLCT character, *increases* at higher dihedral angles.

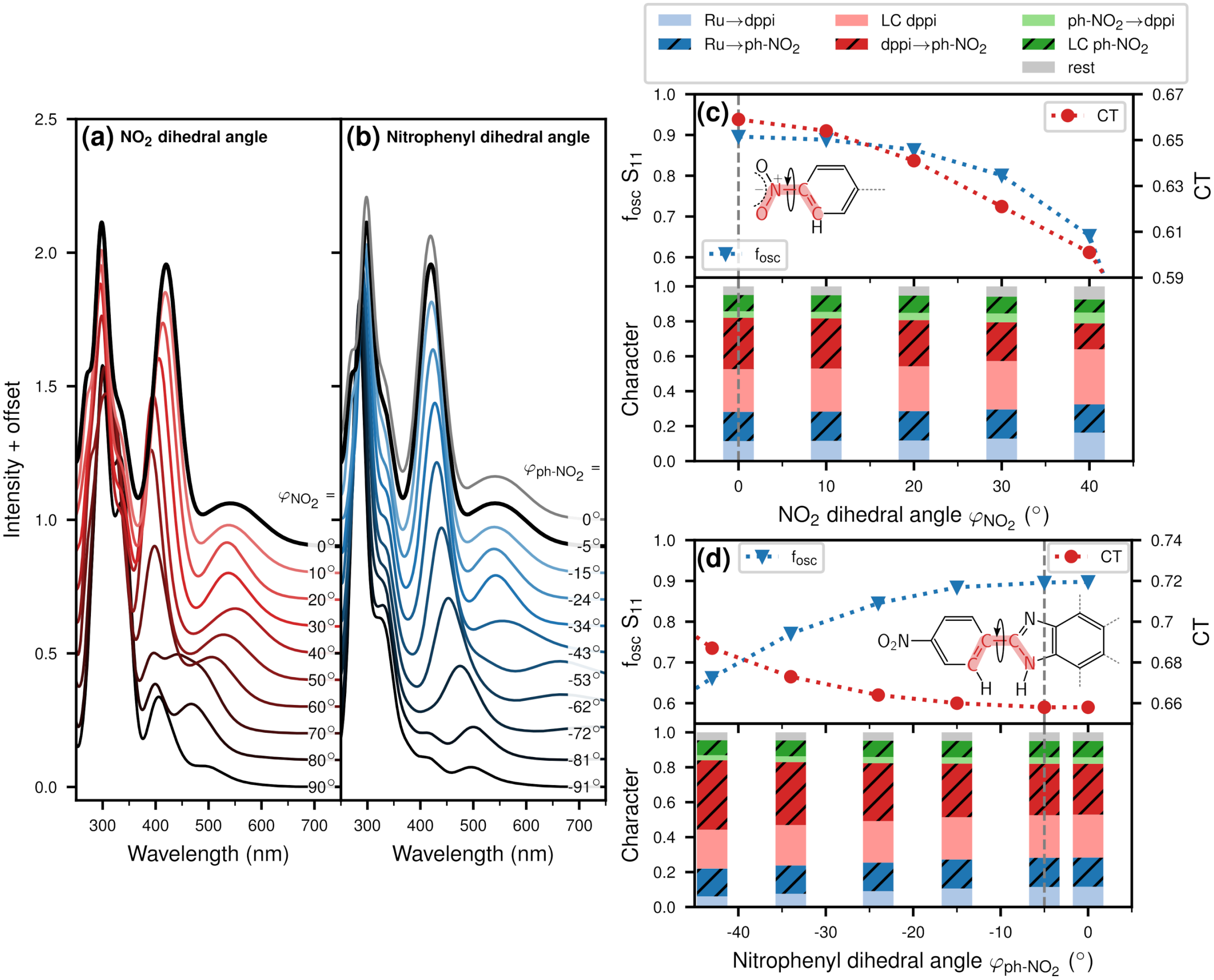

Figure S14: **(a)** Effect of NO_2 and **(b)** nitrophenyl torsion on the absorption spectra of 1H-Ru(dppip-NO_2). The spectra were calculated from structures from relaxed scans of the NO_2 and nitrophenyl dihedral angles, respectively. Influence of **(c)** NO_2 and **(d)** nitrophenyl torsion on the bright vis state (S_{11}) in 1H-Ru(dppip-NO_2) structures from relaxed dihedral angle scans, showing changes in oscillator strength f_{osc}, CT number, and character of S_{11}.

S5.2 Unrelaxed scans

Fig. S15 presents unrelaxed scans along the NO_2 and nitrophenyl dihedral angles in 1H-Ru(dppip-NO_2) (Fig. S15a–b) as well as along the nitrophenyl dihedral angle in 0H,2H-Ru(dppip-NO_2) (Fig. S15b). The estimated torsion barriers are higher than the values from the corresponding relaxed scans (Fig. 6 in the main text), *e.g.*, 0.24 eV compared to 0.22 eV and 0.23 eV in the relaxed scans of the NO_2 and nitrophenyl dihedral angles in 1H-Ru(dppip-NO_2). However, the differences are rather small and both unrelaxed and relaxed scans follow the same trend: the nitrophenyl torsion barrier increases in the order of 2H-Ru(dppip-NO_2) 0.17 eV $<$ 1H-Ru(dppip-NO_2) 0.24 eV $<$ 0H-Ru(dppip-NO_2) 0.34 eV.

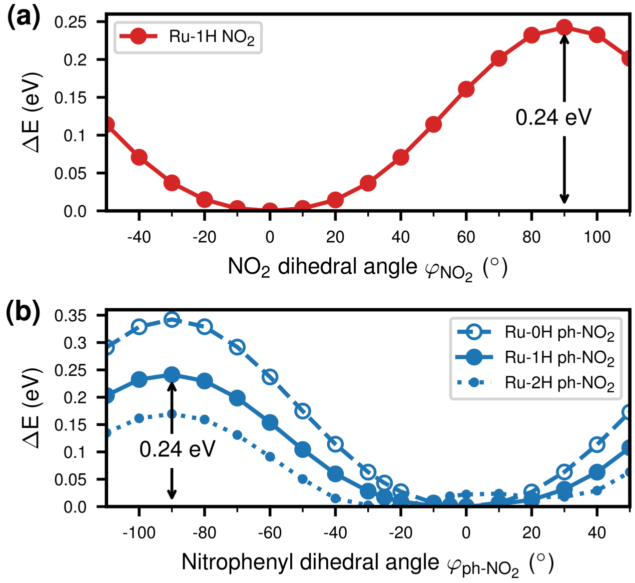

Figure S15: Unrelaxed dihedral angle scans: **(a)** NO_2 dihedral angle φ_{NO_2} in 1H-Ru(dppip-NO_2) and **(b)** nitrophenyl dihedral angle $\varphi_{ph\text{-}NO_2}$ with respect to im–dpp ring system in 0H,1H,2H-Ru(dppip-NO_2).

As no significant differences were observed between the unrelaxed and the relaxed dihedral angle scans, neither in the effect on the absorption spectra and excited state characters in 1H-Ru(dppip-NO_2) structures (Fig. 7 and Fig. S17 compared to Fig. S14) nor in the trend of the torsion barriers (Fig. S15 compared to Fig. 6), we focused on the unrelaxed scans in the following and in the main text.

S5.2.1 0H-Ru(dppip-NO_2)

The absorption spectra of 0H-Ru(dppip-NO_2) structures, where the nitrophenyl dihedral angle was scanned from -0.8° to -50° (unrelaxed scan), are presented in Fig. S16a. Both the vis band at about 460 nm and the low-energy peak below 600 nm are affected by the torsional motion. Fig. S16b shows the effect of the nitrophenyl dihedral angle scan on the oscillator strength and character of the S_{10} state, which is the bright vis state in the 0H-Ru(dppip-NO_2) equilibrium spectrum. The oscillator strength decreases and the CT character increases with the nitrophenyl dihedral angle, which is mainly due to a higher Ru → dppip-NO_2 MLCT character, whereas the LC and LLCT contributions decrease.

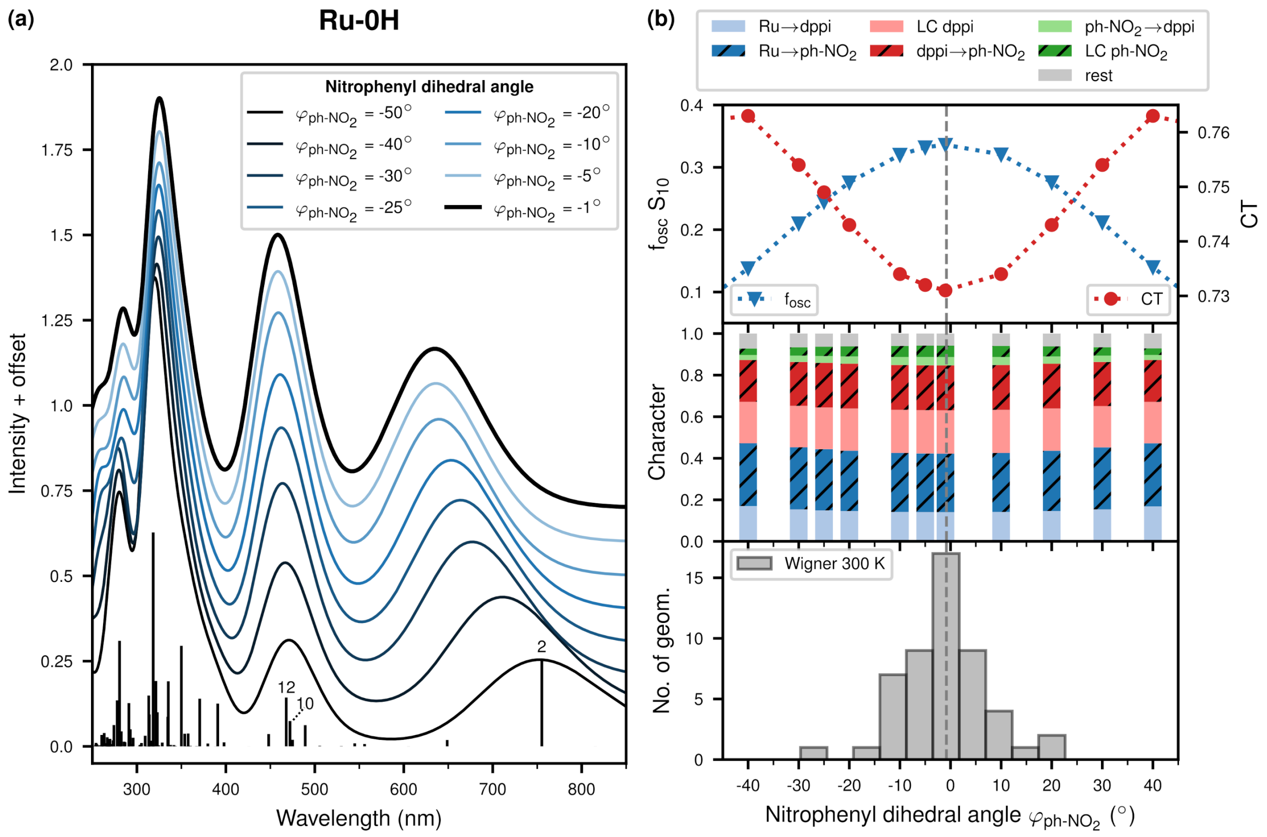

Figure S16: **(a)** Absorption spectra of 0H-Ru(dppip-NO_2) structures, where the nitrophenyl dihedral angle was scanned from -0.8° to -50° (eq.: -0.8°). **(b)** Effect of nitrophenyl torsion on the oscillator strength f_{osc} and character of the bright vis state (S_{10}); the eq. angle is indicated by the dashed gray line. Distribution of dihedral angles in the 300 K Wigner ensemble (50 geometries).

S5.2.2 1H-Ru(dppip-NO_2)

The influence of changes in the dihedral angles on the vis band of 1H-Ru(dppip-NO_2) is presented in Fig. S17. As anticipated, we see significant effects. A decrease in the intensity of the vis band is found for a torsional motion of both the NO_2 group (Fig. S17a) and of the nitrophenyl ring (Fig. S17b). Moreover, both dihedral angle scans show a shift in the computed vis band position. However, it is plausible that the latter is due to changes in the charge transfer *vs.* local character of the bright state, keeping in mind that B3LYP is known to underestimate charge transfer excitation energies.[S17–S19] And yet, in 2H-Ru(dppip-NO_2) an increase of the excitation energy with increasing CT character of the transition is observed at higher nitrophenyl dihedral angles $\varphi_{\text{ph-NO}_2}$ (see below in Fig. S19).

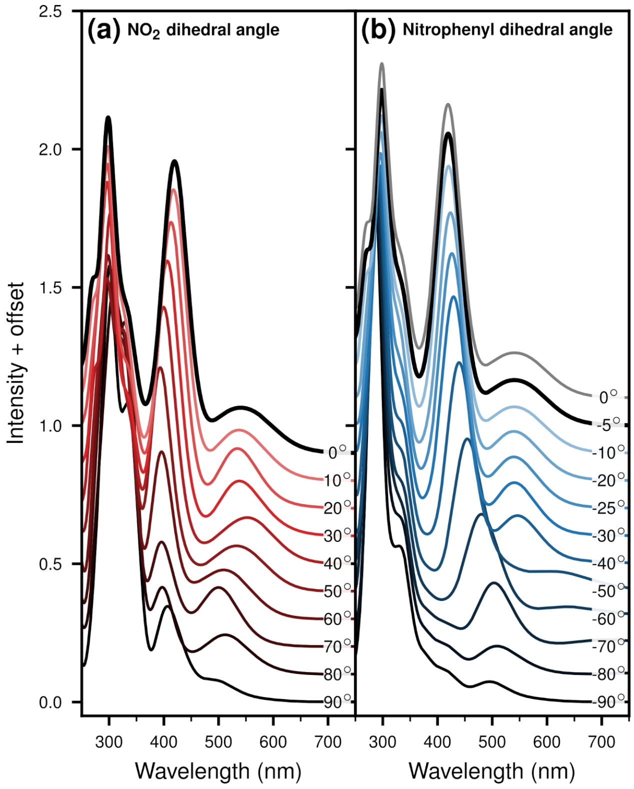

Figure S17: **(a)** Effect of NO_2 and **(b)** nitrophenyl torsion on the absorption spectra of 1H-Ru(dppip-NO_2). The spectra were calculated from structures in which the NO_2 and nitrophenyl dihedral angles were scanned from 0° to 90° or from 0° to -90°, respectively (unrelaxed scans).

The effect of a torsional motion of the NO_2 group and nitrophenyl group (unrelaxed

dihedral angle scans) on the character of the S_{11} state, which dominates the vis absorption band in the equilibrium spectrum of 1H-Ru(dppip-NO_2), was discussed in the main text. The changes in the CT character, or in the localization of the excited electron, with increasing NO_2 and nitrophenyl dihedral angles can also be shown by the matrix plot of the TheoDORE CT numbers of S_{11} of 1H-Ru(dppip-NO_2) in Fig. S18a–b (*cf.* Fig. S1c for an explanation of the matrix plot of the CT numbers). The figure compares dihedral angles of 0°, |40|°, and |90|° to show the effect more clearly. It demonstrates the opposing effects of NO_2 and nitrophenyl torsion. While the former increases the dppi-centered local excitation character and effectively quenches CT excitations to the nitrophenyl group (Fig. S18a), the latter increases the dppi $\rightarrow$ nitrophenyl LLCT character of the excited state and the localization of the electron on the nitrophenyl ring (Fig. S18b).

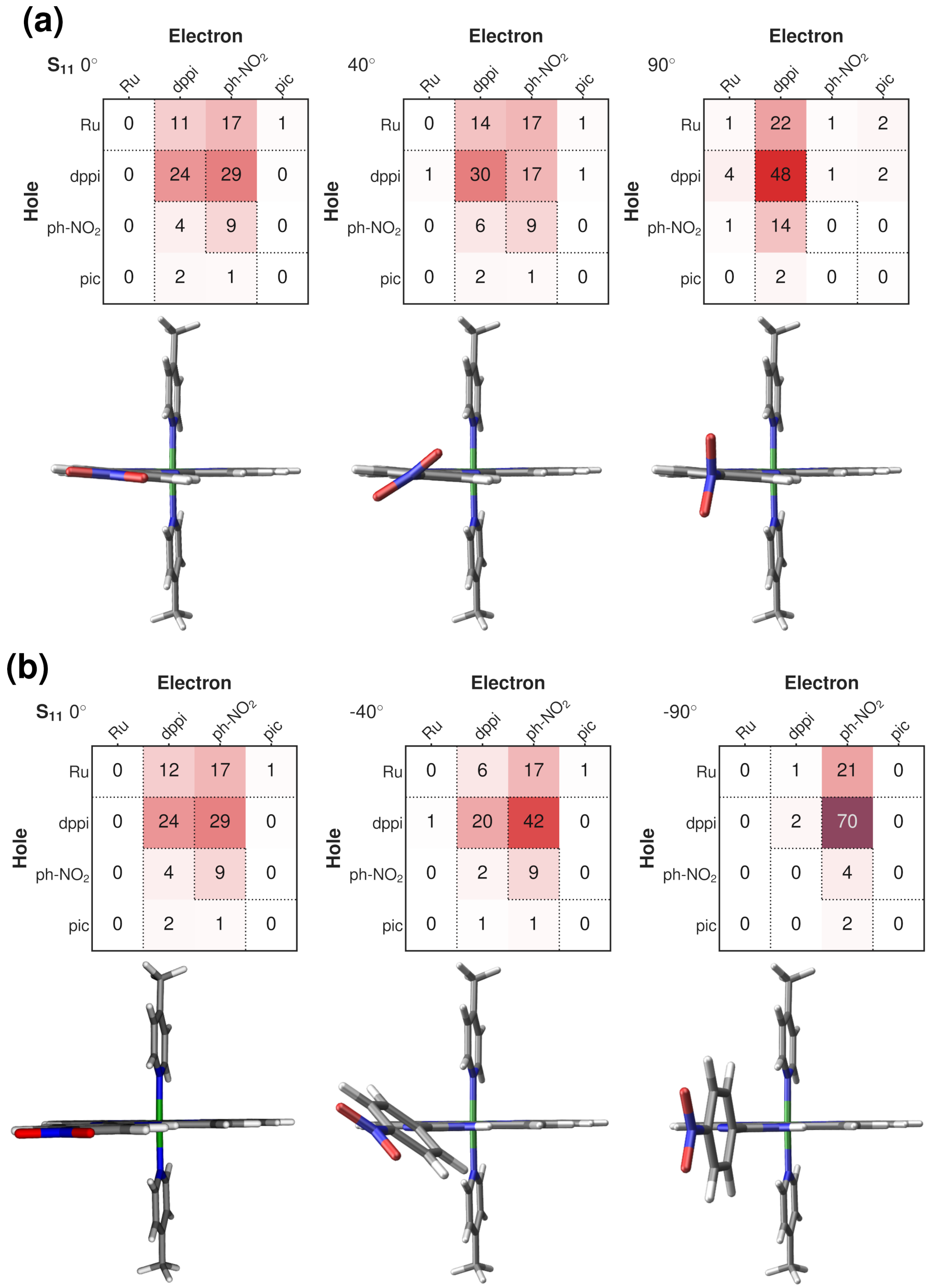

Figure S18: Matrix plot of CT numbers of S_{11} state of 1H-Ru(dppip-NO_2) showing **(a)** the increasing localization of the excited electron on the im–dpp ring (dppi fragment) with increasing NO_2 dihedral angle φ_{NO_2} (dppi LC excitation). In the planar structure (0°), the excited electron is more distributed over the equatorial dppip-NO_2 ligand and there is a greater CT character to the nitrophenyl group. **(b)** Increasing localization of the excited electron on the nitrophenyl ring (ph-NO_2 fragment) with increasing nitrophenyl dihedral angle $\varphi_{\text{ph-NO}_2}$. The corresponding 1H-Ru(dppip-NO_2) structures with varying dihedral angles (φ_{NO_2} or $\varphi_{\text{ph-NO}_2} = 0°, |40|°, |90|°$) are shown below the matrix plots.

S5.2.3 2H-Ru(dppip-NO_2)

Fig. S19a shows the absorption spectra of 2H-Ru(dppip-NO_2) structures, where the nitrophenyl dihedral angle was scanned from 0° to -50° (unrelaxed scans). The effect of the scan on the oscillator strength and character of the S_{13} state, which is the bright vis state in the 2H-Ru(dppip-NO_2) equilibrium spectrum, is presented in Fig. S19b. Like in 0H,1H-Ru(dppip-NO_2), higher dihedral angles lead to a decrease in oscillator strength and increase in the CT character of the excited state. In 2H-Ru(dppip-NO_2), mainly the dppi-centered LC character is reduced, whereas the dppi → nitrophenly LLCT contribution is increased at higher nitrophenyl dihedral angles. However, these changes in the character of S_{13} are small in 2H-Ru(dppip-NO_2).

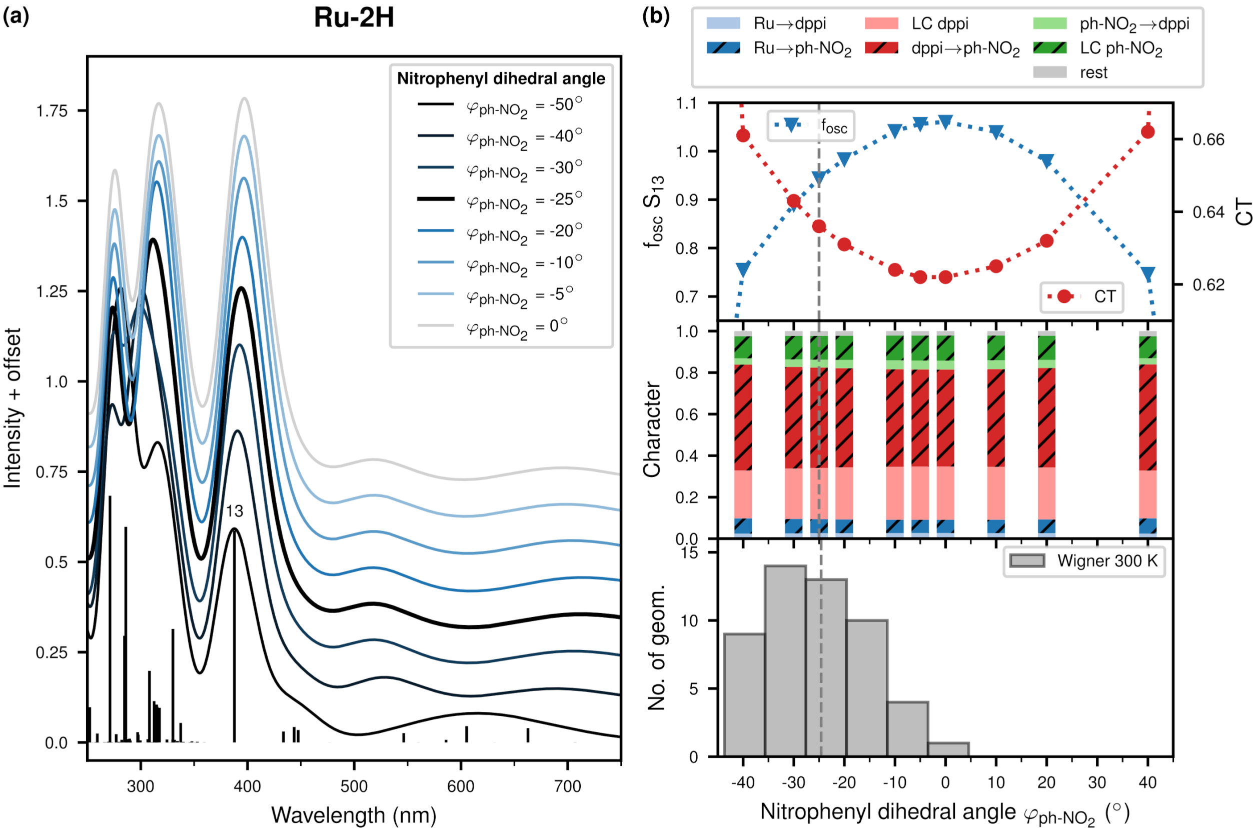

Figure S19: **(a)** Absorption spectra of 2H-Ru(dppip-NO_2) structures, where the nitrophenyl dihedral angle was scanned from 0° to -50° (eq.: -25°). **(b)** Effect of nitrophenyl torsion on the oscillator strength f_{osc} and character of the bright vis state (S_{13}); the eq. angle is indicated by the dashed gray line. Distribution of dihedral angles in the 300 K Wigner ensemble (50 geometries).

S5.2.4 Comparison of 0H,1H,2H-Ru(dppip-NO_2)

Matrix plots of the TheoDORE CT numbers of the bright vis states S_{10}, S_{11}, S_{13} in 0H,1H,2H-Ru(dppip-NO_2) are shown in Fig. S20 for *ca.* planar geometries and for structures with a nitrophenyl dihedral angle of -40°. The excited electron is more distributed over the equatorial dppip-NO_2 ligand in the nearly planar structures compared to the structures with a rotated nitrophenyl ring, where the excited electron is rather localized on the nitrophenyl ring. In the deprotonated 0H-Ru(dppip-NO_2) complex, MLCT excitations are more important than dppi → dppip-NO_2 LLCT/LC excitations. In contrast, dppi → nitrophenyl excitations dominate the character of S_{13} in the protonated 2H-Ru(dppip-NO_2).

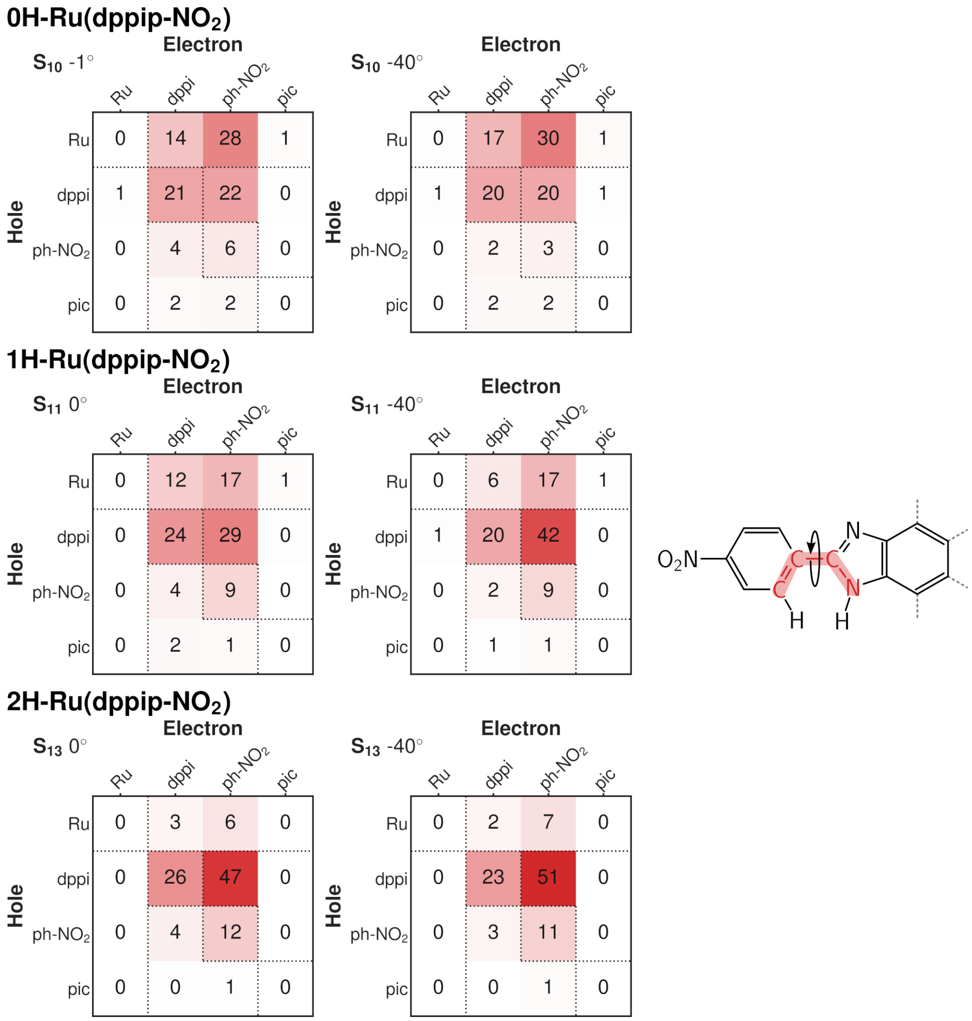

Figure S20: Matrix plot of CT numbers of S_{10}, S_{11}, S_{13} states of 0H,1H,2H-Ru(dppip-NO_2) at nitrophenyl dihedral angles of 0° (*ca.* -1° for 0H-Ru(dppip-NO_2)), and -40°.

S6 Optimized geometries

The Cartesian coordinates of the optimized geometries of the 0H,1H,2H-Ru(dppip-NO_2) complexes and of Ru(dpp) are reported below (B3LYP-D3BJ/def2-TZVP and def2-ECP on Ru, C-PCM acetonitrile, RIJCOSX SARC/J). Frequency calculations based on these geometries were used for generating the Wigner ensembles.

83

Coordinates Ru-0H

Ru	2.243419	0.581302	14.521357
N	3.494587	1.422759	12.950701
N	1.182959	0.306721	12.894074
N	0.695499	-0.289599	15.353012
N	2.764994	0.529361	16.633237
N	1.435142	2.498231	14.827439
N	3.255334	-1.244651	14.279501
N	-3.177693	-1.585546	11.718715
N	-3.612258	-2.117320	13.906196
N	-9.093755	-4.232776	11.081990
C	0.140609	2.797405	14.631957
C	2.249327	3.486493	15.237942
C	1.805136	4.774155	15.463244
C	0.461520	5.095853	15.267897
C	-0.371871	4.064447	14.838448
C	2.910778	1.325148	11.714320
C	3.550310	1.808949	10.580467
C	4.800006	2.401661	10.697509
C	5.384743	2.497882	11.951656
C	4.696420	1.995734	13.047284
C	1.584729	0.686131	11.682278
C	-0.007873	-0.297923	13.110167
C	-0.905359	-0.566944	12.066299
C	-0.488704	-0.171083	10.773952
C	0.736041	0.445255	10.584582
C	-0.279322	-0.629986	14.478596
C	-1.466135	-1.252686	14.892862
C	-2.394839	-1.536277	13.838747
C	-2.123344	-1.204069	12.471282

C	-4.039897	-2.124508	12.617789
C	-1.580055	-1.506907	16.279518
C	-0.565182	-1.147312	17.149480
C	0.598020	-0.521307	16.661046
C	-5.333099	-2.667520	12.225812
C	-6.195218	-3.232120	13.180820
C	-7.423201	-3.745355	12.813630
C	-7.803398	-3.697492	11.474006
C	-6.970395	-3.145096	10.503094
C	-5.744780	-2.635472	10.882664
C	1.785352	-0.052912	17.395317
C	3.867099	0.976284	17.239839
C	4.068460	0.878930	18.609566
C	3.081401	0.290799	19.386613
C	1.929349	-0.178825	18.770859
C	-0.061999	6.474669	15.530100
C	2.651414	-2.379246	13.890347
C	3.330561	-3.574246	13.745011
C	4.698755	-3.638296	14.003351
C	5.314838	-2.453932	14.409279
C	4.574826	-1.295006	14.533582
C	5.474235	-4.908327	13.831362
O	-9.816939	-4.718293	11.949780
O	-9.415988	-4.180359	9.896665
H	5.117087	2.051446	14.042460
H	6.355925	2.951868	12.090418
H	5.306328	2.781885	9.820228
H	3.071564	1.721463	9.615584
H	1.038769	0.743697	9.590742
H	-1.135209	-0.350968	9.925878
H	-2.470627	-1.985881	16.662980
H	-0.669573	-1.348199	18.206344
H	1.145280	-0.641253	19.353366
H	3.202693	0.197041	20.457604
H	4.981585	1.259137	19.045667
H	4.612171	1.427909	16.598274
H	3.287187	3.226050	15.385085
H	2.512552	5.525564	15.788869

H	-1.424320	4.241137	14.658970
H	-0.503073	1.997954	14.297354
H	5.040363	-0.372676	14.848680
H	6.373211	-2.429356	14.633731
H	2.784203	-4.454488	13.432313
H	1.591399	-2.327588	13.692859
H	-0.934625	6.688741	14.913058
H	-0.365893	6.565140	16.577160
H	0.703345	7.228094	15.343156
H	4.840406	-5.781181	13.987791
H	5.873862	-4.966412	12.814609
H	6.318407	-4.949946	14.519869
H	-5.087881	-2.203333	10.141771
H	-7.286751	-3.119421	9.471277
H	-8.085317	-4.178751	13.547869
H	-5.887671	-3.262460	14.215891

84

Coordinates Ru-1H

Ru	2.256232	0.599088	14.479334
N	2.703867	0.712270	16.604866
N	0.661192	-0.160044	15.326554
N	1.243951	0.213156	12.846103
N	3.580315	1.268242	12.886381
N	1.498699	2.558422	14.595005
N	3.228943	-1.265492	14.435354
N	-3.639580	-2.029416	13.900592
N	-3.143273	-1.706133	11.756589
N	-9.148893	-4.320058	11.272730
C	1.688117	0.215162	17.379319
C	1.784764	0.196774	18.763459
C	2.928539	0.690368	19.377282
C	3.951672	1.193343	18.588411
C	3.796285	1.184361	17.208850
C	0.513079	-0.285586	16.643846
C	-0.293063	-0.552948	14.449848
C	-1.500596	-1.116169	14.874587
C	-1.673102	-1.256790	16.268303

C	-0.678615	-0.846913	17.140402
C	-2.398471	-1.471449	13.812328
C	-2.062589	-1.258214	12.459758
C	-0.830633	-0.684619	12.020052
C	0.031637	-0.344474	13.070454
C	1.693646	0.473907	11.619589
C	0.877198	0.157437	10.517602
C	-0.368305	-0.413732	10.712595
C	3.036042	1.080017	11.642558
C	4.794630	1.814429	12.975852
C	5.532297	2.201356	11.865035
C	4.986964	2.013552	10.604324
C	3.724364	1.446081	10.494537
C	2.331437	3.554826	14.943786
C	1.921272	4.869313	15.043180
C	0.594567	5.211034	14.779445
C	-0.258625	4.170040	14.416615
C	0.219697	2.876023	14.336587
C	0.106736	6.621535	14.903905
C	2.613517	-2.414686	14.113153
C	3.266926	-3.632313	14.097909
C	4.620278	-3.704939	14.423780
C	5.248130	-2.505077	14.759849
C	4.533873	-1.323658	14.754406
C	5.369154	-5.001576	14.392226
C	-4.073371	-2.163001	12.653952
C	-5.367725	-2.714669	12.275505
C	-5.801590	-2.726733	10.943474
C	-7.036883	-3.252419	10.610668
C	-7.840538	-3.766696	11.620089
C	-7.437048	-3.767795	12.950775
C	-6.201005	-3.241324	13.272596
O	-9.847296	-4.766557	12.175026
O	-9.487601	-4.312443	10.095270
H	5.185079	1.943769	13.976431
H	6.511601	2.639118	11.998642
H	5.532708	2.302970	9.716235
H	3.275628	1.288174	9.524200

H	1.219969	0.360845	9.513157
H	-0.987528	-0.651791	9.858215
H	-2.586450	-1.686421	16.655849
H	-0.821996	-0.959543	18.205515
H	0.972696	-0.201054	19.355120
H	3.014604	0.680474	20.455635
H	4.859443	1.588436	19.022405
H	4.571068	1.567568	16.558201
H	3.356270	3.280116	15.145404
H	2.642393	5.625448	15.324577
H	-1.299830	4.360099	14.191346
H	-0.440543	2.069985	14.054583
H	5.009563	-0.389831	15.015615
H	6.295601	-2.485251	15.030882
H	2.712017	-4.522965	13.833675
H	1.564973	-2.358083	13.863068
H	-0.726977	6.810950	14.227809
H	-0.245714	6.804663	15.923328
H	0.905627	7.333931	14.698234
H	4.707692	-5.843564	14.596005
H	5.805372	-5.155298	13.400718
H	6.185328	-5.002338	15.114769
H	-3.226026	-1.715554	10.751098
H	-5.185706	-2.320442	10.153450
H	-7.376295	-3.262274	9.586272
H	-8.082220	-4.174853	13.714284
H	-5.868402	-3.234212	14.300180

85

Coordinates Ru-2H

Ru	2.250451	0.609630	14.488055
N	3.566097	1.254206	12.880284
N	1.226968	0.212075	12.867543
N	0.659589	-0.133832	15.352632
N	2.713225	0.740567	16.607438
N	1.511984	2.577749	14.589709
N	3.219924	-1.258655	14.459790
N	-3.157938	-1.734555	11.807758

N	-3.632618	-2.034939	13.892393
N	-9.076180	-4.491204	11.172188
C	0.234721	2.906238	14.336746
C	2.358450	3.568584	14.920906
C	1.963616	4.888490	15.007167
C	0.639040	5.241872	14.748256
C	-0.228535	4.206202	14.404417
C	3.013887	1.055846	11.641457
C	3.695748	1.404689	10.485140
C	4.962521	1.966269	10.581119
C	5.515931	2.164650	11.836134
C	4.783522	1.794382	12.956615
C	1.668280	0.456001	11.633991
C	0.014465	-0.340862	13.104256
C	-0.848721	-0.691477	12.061751
C	-0.400843	-0.437788	10.748343
C	0.844649	0.129590	10.540921
C	-0.301051	-0.534299	14.487243
C	-1.502194	-1.096646	14.931376
C	-2.382757	-1.459539	13.863844
C	-2.076574	-1.266170	12.516763
C	-4.097902	-2.201187	12.644497
C	-1.667263	-1.227949	16.326204
C	-0.665005	-0.808224	17.184002
C	0.521126	-0.250288	16.673021
C	-5.373515	-2.784418	12.267964
C	-6.016566	-3.675210	13.135867
C	-7.230255	-4.234452	12.779579
C	-7.785536	-3.895824	11.553894
C	-7.168309	-3.017059	10.675182
C	-5.955768	-2.458429	11.037056
C	1.702658	0.253474	17.395088
C	3.811667	1.213906	17.198256
C	3.977028	1.233689	18.577064
C	2.959354	0.741038	19.378533
C	1.808882	0.245740	18.777995
C	0.168529	6.659231	14.857650
C	2.602086	-2.411112	14.154264

C	3.253468	-3.629582	14.153628
C	4.607641	-3.699730	14.477108
C	5.238230	-2.496317	14.794564
C	4.525806	-1.314043	14.775315
C	5.353824	-4.997930	14.462524
O	-9.608400	-5.258384	11.960740
O	-9.548486	-4.187369	10.086734
H	5.181345	1.932612	13.952992
H	6.498495	2.598221	11.958877
H	5.504581	2.242657	9.686755
H	3.240411	1.238508	9.519301
H	1.181564	0.321100	9.532335
H	-1.026555	-0.684856	9.901557
H	-2.573516	-1.656860	16.731644
H	-0.799930	-0.913212	18.250734
H	1.000535	-0.144679	19.379550
H	3.054209	0.739998	20.456089
H	4.889541	1.629321	19.000455
H	4.583033	1.588582	16.538801
H	3.381821	3.285943	15.118476
H	2.695196	5.639640	15.274391
H	-1.269107	4.405280	14.184345
H	-0.437195	2.104995	14.068977
H	5.004399	-0.378032	15.022721
H	6.286538	-2.474192	15.061946
H	2.696586	-4.522794	13.902691
H	1.553011	-2.358138	13.905799
H	-0.673704	6.847282	14.191956
H	-0.165358	6.861791	15.879678
H	0.971987	7.359324	14.628887
H	4.692699	-5.834796	14.687329
H	5.780009	-5.170193	13.469661
H	6.176934	-4.986982	15.176941
H	-3.220988	-1.775121	10.798986
H	-5.482236	-1.754087	10.367323
H	-7.631919	-2.770193	9.732553
H	-7.734468	-4.927117	13.435469
H	-5.565109	-3.955332	14.077578

H	-4.152392	-2.261999	14.730463

69

Coordinates Ru(dpp)

Ru	-0.069433	-0.540387	16.564127
N	0.452990	-2.482304	17.419970
N	0.887048	-1.458925	15.120808
N	-0.183076	0.883644	15.222523
N	-1.157859	1.062204	17.576555
N	-1.921060	-1.355884	15.988849
N	1.711210	0.227187	17.378074
C	1.141765	-3.276585	16.539609
C	1.563243	-4.549086	16.897249
C	1.284646	-5.030258	18.169985
C	0.588229	-4.223043	19.055892
C	0.192823	-2.958832	18.638791
C	1.392215	-2.684380	15.213610
C	0.996575	-0.721616	13.989374
C	1.640711	-1.185823	12.842575
C	2.184028	-2.491636	12.930677
C	2.061630	-3.227011	14.096264
C	1.673016	-0.287912	11.721936
C	1.102037	0.956364	11.775177
C	0.438174	1.436279	12.955017
C	0.406367	0.566147	14.044712
C	-0.777623	2.055772	15.417970
C	-0.789640	2.993080	14.363373
C	-0.192117	2.689816	13.152905
C	-1.341852	2.159762	16.775290
C	-1.642341	1.092359	18.819487
C	-2.326390	2.184281	19.337439
C	-2.517180	3.295515	18.531044
C	-2.017699	3.280746	17.235211
C	-2.786011	-1.737892	16.944828
C	-4.020430	-2.285571	16.657763
C	-4.416340	-2.464636	15.331937
C	-3.513184	-2.060516	14.349984
C	-2.293392	-1.519352	14.708961

C	-5.735288	-3.082022	14.982047
C	2.714081	0.723044	16.635516
C	3.878236	1.219812	17.190329
C	4.050821	1.218380	18.573496
C	3.000558	0.704173	19.334775
C	1.864705	0.224359	18.713986
C	5.309909	1.720366	19.210448
H	-1.473154	0.206688	19.417319
H	-2.696882	2.151411	20.352431
H	-3.046027	4.163848	18.900608
H	-2.151996	4.133766	16.585376
H	-1.266816	3.953086	14.499681
H	-0.206180	3.415573	12.350272
H	1.147283	1.604048	10.908938
H	2.164561	-0.613746	10.814124
H	2.698557	-2.916958	12.078917
H	2.481282	-4.221484	14.148338
H	2.103755	-5.157229	16.185995
H	1.608263	-6.020788	18.460531
H	0.348764	-4.555615	20.056199
H	-0.353125	-2.299455	19.300147
H	-2.471890	-1.592520	17.967976
H	-4.672602	-2.568857	17.473470
H	-3.754418	-2.161826	13.299975
H	-1.593834	-1.205389	13.949226
H	1.045868	-0.175920	19.293511
H	3.061162	0.677046	20.414870
H	4.647451	1.609337	16.536472
H	2.578845	0.723743	15.564635
H	-6.098789	-2.716078	14.021808
H	-5.628684	-4.168024	14.903680
H	-6.481456	-2.878481	15.750101
H	5.786520	2.482653	18.594331
H	5.113119	2.130202	20.201201
H	6.020788	0.897209	19.328697

References

(S1) Plasser, F. TheoDORE: A package for theoretical density, orbital relaxation, and exciton analysis. http://theodore-qc.sourceforge.net, 2019; http://theodore-qc.sourceforge.net.

(S2) Plasser, F. TheoDORE: A toolbox for a detailed and automated analysis of electronic excited state computations. *J. Chem. Phys.* **2020**, *152*, 084108.

(S3) Plasser, F.; Wormit, M.; Dreuw, A. New tools for the systematic analysis and visualization of electronic excitations. I. Formalism. *J. Chem. Phys.* **2014**, *141*, 024106.

(S4) Mai, S.; Plasser, F.; Dorn, J.; Fumanal, M.; Daniel, C.; González, L. Quantitative wave function analysis for excited states of transition metal complexes. *Coord. Chem. Rev.* **2018**, *361*, 74–97.

(S5) Yanai, T.; Tew, D. P.; Handy, N. C. A new hybrid exchange–correlation functional using the Coulomb-attenuating method (CAM-B3LYP). *Chem. Phys. Lett.* **2004**, *393*, 51–57.

(S6) Plasser, F.; Ruckenbauer, M.; Mai, S.; Oppel, M.; Marquetand, P.; González, L. Efficient and Flexible Computation of Many-Electron Wave Function Overlaps. *J. Chem. Theory Comput.* **2016**, *12*, 1207–1219.

(S7) Huber, F. L.; Wernbacher, A. M.; Perleth, D.; Nauroozi, D.; González, L.; Rau, S. A Ruthenium(II) Water Oxidation Catalyst Containing a pH Responsive Ligand Framework. *Inorg. Chem.* **2021**, Accepted Manuscript. DOI: 10.1021/acs.inorgchem.1c01646.

(S8) Kupfer, S.; Guthmuller, J.; Wächtler, M.; Losse, S.; Rau, S.; Dietzek, B.; Popp, J.; González, L. Protonation effects on the resonance Raman properties of a novel

(terpyridine)Ru(4H-imidazole) complex: an experimental and theoretical case study. *Phys. Chem. Chem. Phys.* **2011**, *13*, 15580–15588.

(S9) Coe, B. J.; Pilkington, R. A. Theoretical Studies on Two-Dimensional Nonlinear Optical Chromophores with Pyrazinyl Cores and Organic or Ruthenium(II) Ammine Electron Donors. *J. Phys. Chem. A* **2014**, *118*, 2253–2268.

(S10) Jäger, M.; Freitag, L.; González, L. Using computational chemistry to design Ru photosensitizers with directional charge transfer. *Coord. Chem. Rev.* **2015**, *304-305*, 146–165.

(S11) Sánchez-Murcia, P. A.; Nogueira, J. J.; González, L. Exciton Localization on Ru-Based Photosensitizers Induced by Binding to Lipid Membranes. *J. Phys. Chem. Lett.* **2018**, *9*, 683–688.

(S12) de Souza, B.; Farias, G.; Neese, F.; Izsák, R. Predicting Phosphorescence Rates of Light Organic Molecules Using Time-Dependent Density Functional Theory and the Path Integral Approach to Dynamics. *J. Chem. Theory Comput.* **2019**, *15*, 1896–1904.

(S13) Zong, R.; Thummel, R. P. 2,9-Di-(2‘-pyridyl)-1,10-phenanthroline: A Tetradentate Ligand for Ru(II). *J. Am. Chem. Soc.* **2004**, *126*, 10800–10801.

(S14) Zhang, G.; Zong, R.; Tseng, H.-W.; Thummel, R. P. Ru(II) Complexes of Tetradentate Ligands Related to 2,9-Di(pyrid-2‘-yl)-1,10-phenanthroline. *Inorg. Chem.* **2008**, *47*, 990–998.

(S15) Muckerman, J. T.; Kowalczyk, M.; Badiei, Y. M.; Polyansky, D. E.; Concepcion, J. J.; Zong, R.; Thummel, R. P.; Fujita, E. New Water Oxidation Chemistry of a Seven-Coordinate Ruthenium Complex with a Tetradentate Polypyridyl Ligand. *Inorg. Chem.* **2014**, *53*, 6904–6913.

(S16) Mai, S.; Gattuso, H.; Monari, A.; González, L. Novel Molecular-Dynamics-Based Protocols for Phase Space Sampling in Complex Systems. *Front. Chem.* **2018**, *6*, 495.

(S17) Tozer, D. J.; Amos, R. D.; Handy, N. C.; Roos, B. O.; Serrano-Andres, L. Does density functional theory contribute to the understanding of excited states of unsaturated organic compounds? *Mol. Phys.* **1999**, *97*, 859–868.

(S18) Dreuw, A.; Head-Gordon, M. Failure of Time-Dependent Density Functional Theory for Long-Range Charge-Transfer Excited States: The Zincbacteriochlorin–Bacteriochlorin and Bacteriochlorophyll–Spheroidene Complexes. *J. Am. Chem. Soc.* **2004**, *126*, 4007–4016.

(S19) Daniel, C. Photochemistry and photophysics of transition metal complexes: Quantum chemistry. *Coord. Chem. Rev.* **2015**, *282-283*, 19–32.
